# Determinants of outcomes for acute encephalopathy with reduced subcortical diffusion

**DOI:** 10.1038/s41598-020-66167-7

**Published:** 2020-06-04

**Authors:** Kensuke Sakata, Go Kawano, Masao Suda, Takaoki Yokochi, Yukako Yae, Toru Imagi, Yukihiro Akita, Keizo Ohbu, Toyojiro Matsuishi

**Affiliations:** 1 Department of Paediatrics, St Mary’s Hospital, 422 Tsubukuhonmachi, Kurume, Fukuoka 830-8543 Japan; 2 Department of Paediatrics, Kurume University Hospital, 67 Asahimachi, Kurume, Fukuoka 830-0011 Japan; 3 Research Centre for Children and Research Centre for Rett Syndrome, St Mary’s Hospital, 422 Tsubukuhonmachi, Kurume, Fukuoka 830-8543 Japan

**Keywords:** Encephalopathy, Paediatric research, Encephalopathy

## Abstract

Acute encephalopathy with reduced subcortical diffusion (AED), characterised by seizure onset and widespread reduced apparent diffusion coefficient in the cortex/subcortical white matter, is one of the most common acute encephalopathies in children in East Asia. This 14-year single-centre retrospective study on 34 patients with AED showed that therapeutic hypothermia was used for patients with more severe consciousness disturbance after the first seizure or second phase initiation, extrapolating from neonatal hypoxic encephalopathy and adult post-cardiac arrest syndrome. The basal ganglia/thalamus lesions and the Tada score were the poor outcome determinants in the multivariate analysis. The correlation between the worse outcomes and the duration from the first seizure to the initiation of therapeutic hypothermia was observed only in the patients with AED cooled before the second phase. This correlation was not observed in the overall AED population. There was a moderate negative association between the worse outcomes and the duration between the first seizure and the second phase. Therefore, the basal ganglia/thalamus lesions and the Tada score were the outcome determinants for patients with AED. Further investigation is required to examine the efficacy of therapeutic hypothermia in this population while considering the timing of the therapeutic hypothermia initiation and the second phase.

## Introduction

Acute encephalopathy with reduced subcortical diffusion (AED) is one of the most common childhood acute encephalopathies in East Asia. AED was defined by Hayashi as an acute encephalopathy type presenting with (1) seizure onset and (2) widespread reduced apparent diffusion coefficient (ADC) on brain magnetic resonance imaging (MRI), in the cortex and/or subcortical white matter involving unilateral or bilateral hemispheres^1^. The first seizure is mostly associated with a febrile infection. Reduced ADC in diffuse white matter lesions, primarily affecting the subcortical white matter, is referred to as bright tree appearance on diffusion weighted images (DWI) and appears within 9 days of the first seizure. Usually, no abnormal MRI findings are observed within a few days from the first seizure^2^. Although the underlying mechanisms remain to be fully elucidated, an association with delayed neuronal cell death triggered by extracellular glutamate stimulation has been proposed, as the underlying cause for the first seizure^3^. AED encompasses a spectrum and includes the acute encephalopathy with biphasic seizures and late reduced diffusion, which was originally proposed by Takanashi, and only includes patients with intractable and repetitive seizures, and deteriorating consciousness levels (second phase) after a transient improvement in consciousness post febrile status epilepticus (first phase)^3,4^. The outcomes of patients with AED are mild than in patients with other acute encephalopathy types, such as acute necrotising encephalopathy, haemorrhagic shock, and encephalopathy syndrome^5,6^. Nevertheless, the majority of the affected children present permanent neurological deficits^5^.

Diffuse lesions with an injury around the perirolandic regions, prolonged seizures at onset, coma, or involuntary movements have been associated with poor outcomes in AED according to previous retrospective studies^1,2,7^. Patients with AED without an injury around the perirolandic regions, which has been referred to as central sparing in previous reports, represent a relatively mild phenotype of acute encephalopathy^2^. The presence of basal ganglia lesions is associated with severe outcomes^7^.

Therapeutic hypothermia has been used for managing brain oedema in paediatric patients with brain injuries, in several Japanese tertiary centres including our facility, including post-cardiac arrest syndrome and acute encephalopathy associated with febrile infection, which has been extrapolated from neonatal hypoxic encephalopathy and adult post-cardiac arrest syndrome^8–11^. However, there is still a paucity of evidence-based treatments for this disease, including therapeutic hypothermia, and the 2016 Japanese practice guideline for acute encephalopathy in childhood^12^ did not recommend any treatment other than conventional respiratory, haemodynamic management, and seizure control.

To examine the determinants of disease outcomes in patients with AED, we performed a retrospective study on 34 such patients admitted to our facility over a period of 14 years. Therapeutic hypothermia was performed for those who were in a more severe condition, following our institutional protocol for managing patients with consciousness disturbance.

## Results

### Study population

**Figure 1:**
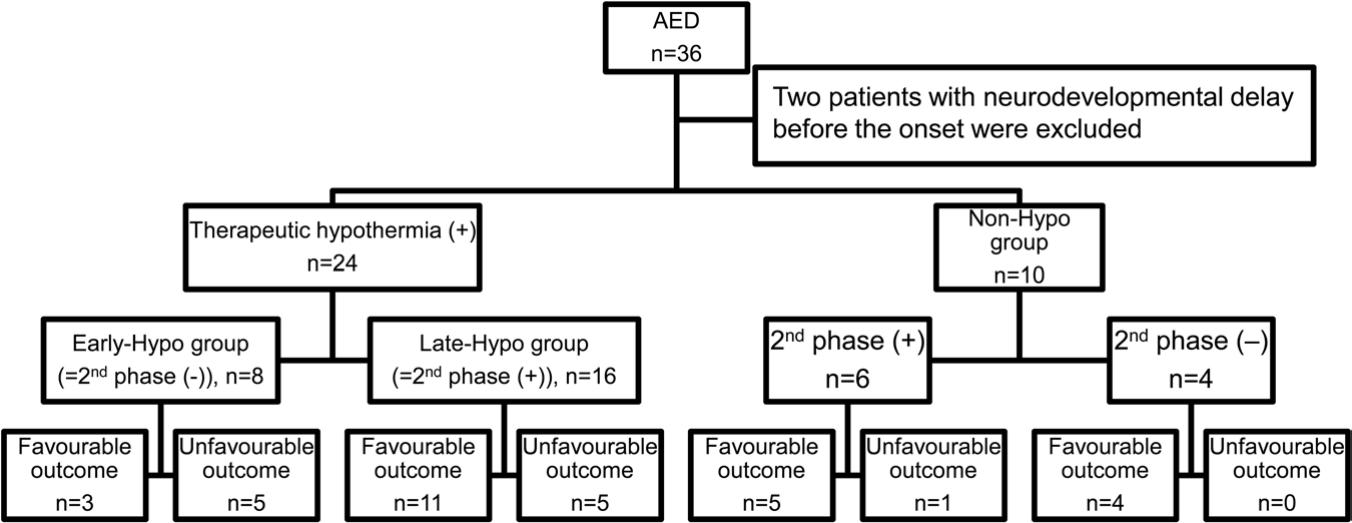
Study population and outcomes. Among 36 children with acute encephalopathy, 34 were in this study enrolled after excluding two patients with AED with a neurodevelopmental delay before the onset. Therapeutic hypothermia was provided to 24 patients, of which eight and 16 patients were cooled before (Early-Hypo group) and after the initiation of the second phase (Late-Hypo group). Therapeutic hypothermia was not provided to 10 patients, of which six and four patients had and did not have the second phase, respectively. Three out of eight patients in the Early-Hypo group had a favourable outcome. Eleven out of 16 patients in the Late-Hypo group had a favourable outcome. In the Non-Hypo group, five out of six patients with the second phase had a favourable outcome and all four patients without the second phase had a favourable outcome. AED, acute encephalopathy with reduced subcortical diffusion.

We identified 34 patients with AED (sex, 18 female and 16 male; mean age [months ± standard deviation], 27.7 ± 24.1 months), after excluding two patients with a neurodevelopmental delay before onset, from the total 36 patients admitted to our facility from February 2004 and August 2018. The febrile seizure was the first neurological symptom in all patients with AED. Eight patients who underwent therapeutic hypothermia by the attending physicians before the second phase, following our institutional protocol for managing patients with consciousness disturbance (Supplementary Data) were included in the Early-Hypo group. Sixteen patients who started therapeutic hypothermia after the initiation of the second phase were included in the Late-Hypo group (Fig. 1). Ten patients who were not provided therapeutic hypothermia were included in the Non-Hypo group. No patients in the Early-Hypo and Late-Hypo groups exhibited seizures during the therapeutic hypothermia. The Glasgow Coma Scale (GCS) at 12–24 h after the first seizure was worse in the Early-Hypo than in the Late-Hypo and the Non-Hypo groups (Supplementary Table S1a). The worst GCS, after 24 h from the first seizure was lower in the Late-Hypo than that in the Non-Hypo group (Supplementary Table S1b). There were more patients with the second phase in the Late-Hypo than in the Non-Hypo group. Six of the 10 patients in the Non-Hypo group had a second phase and the worst GCS was more than 12, after 24 h from the first seizure (Supplementary Table S1a,b, Supplementary Table S2a,d, Fig. 1). The number of patients with catecholamine use, thrombocytopenia, or hypokalaemia was lower in the Non-Hypo than in the other two groups (Supplementary Table S1b). Catecholamine use and hypotension were not present in all patients at the time of admission to our facility (Supplementary Table S2b,e).

The cerebral spinal fluid (CSF) was examined in 28 out of 34 patients with AED (Supplementary Table S2c,f). Two patients had abnormal findings; one had slightly elevated cell count (9/μL, Patient No. 25) and the other had slightly elevated protein levels (51 mg/dL, Patient No. 18) in the first CSF examination in addition of a patient with septic meningitis (Patient No. 5). Dexmedetomidine was used in eight patients during therapeutic hypothermia. Two out of eight patients with dexmedetomidine use during cooling had hypotension, while seven out of 16 patients without dexmedetomidine use during cooling had hypotension. None of the patients had a cardiac arrest throughout the disease course.

Additionally, the extra survey for all children with acute encephalopathy treated in our facility during this study period revealed that all the patients (patients with AED and patients with other acute encephalopathy types) who received therapeutic hypothermia had abnormal MRI findings and that there were no patients without abnormal findings on MRI after therapeutic hypothermia (Supplementary Fig. S7).

### Neuroimaging findings among the study population

The distribution of brain lesions on DWI on MRI was in hemispheres, bilateral frontal lobes, and others in 12, seven, and 15 patients, respectively (Supplementary Table S1a, Supplementary Fig. S1–S3). Three patients exhibited diffuse lesions with an injury around the perirolandic regions on DWI in a hemisphere. There were two patients with diffuse lesions without injury around the perirolandic regions on DWI that were observed within the first 3 days from onset (Supplementary Table S2a,d, Patient No. 4 and 26). None of the patients exhibited bilateral diffuse lesions with an injury around the bilateral perirolandic regions (Supplementary Fig. S4) or lesions widening on the second MRI after the first abnormal MRI. Thirteen patients showed basal ganglia/thalamus lesions on MRI (Supplementary Table S1a, Supplementary Fig. S5). One patient showed a midsagittal line shift on the neuroimaging with symptoms, such as anisocoria and coma, at the beginning of the second phase (Patient No. 1, Supplementary Fig. S6).

Subcortical reduced ADC was detected at a median of 8, 4, and 6.5 days from the first seizure in the Early-Hypo, Late-Hypo, and Non-Hypo groups, respectively. The timing of the abnormal MRI was earlier in the Late-Hypo than in the Early-Hypo or the Non-Hypo group. The timing of the abnormal MRI in the Early-Hypo group was the last among the three groups, as there were no abnormal findings on the first MRI in all but one case (Supplementary Table S2a, Patient No. 14; Supplementary Figs. S8 and S9); these cases had begun therapeutic hypothermia after the first MRI, thus, delaying the timing of the second MRI acquisition.

### Intergroup analysis between the favourable and unfavourable outcome groups

The age was significantly lower in the unfavourable than that in the favourable outcome group (*p* = 0.014, Table 1). The percentage of patients with basal ganglia/thalamus lesions on MRI tended to be higher in the unfavourable than in the favourable outcome group; however, this did not reach statistical significance (*p* = 0.06, Table 1). The Tada score, which is a predictive score for acute encephalopathy with biphasic seizures and late reduced diffusion among patients with febrile seizure in the acute phase^13^, was significantly higher in the unfavourable than in the favourable group (*p* = 0.045). Time_1st-2nd_ among patients with the second phase in the unfavourable group was significantly shorter than that in the favourable group (*p* = 0.005, Table 1). The unfavourable group included more patients with therapeutic hypothermia, although it was not statistically significant (*p* = 0.113, Table 1). The percentage of patients requiring catecholamine administration or with thrombocytopenia (<150,000/μL) was higher in the unfavourable than in the favourable outcome group (*p* = 0.026 and 0.030, respectively, Table 2). There was also a significant difference in the minimal platelet count within 10 days after the first seizure between the favourable and the unfavourable outcome groups (*p* = 0.007, Table 2). However, there was no significant difference in the minimal platelet count when the cooling period was excluded (*p* = 1.000, Table 2). There were also no other differences in other background clinical variables including intravenous methylprednisolone pulse therapy, immunoglobulin administration, or dexmedetomidine use during cooling, between the two groups.

**Table 1:** Comparison of clinical features between favourable and unfavourable outcome groups.

	Favourable outcome			Unfavourable outcome			Effect size	p value
	(PCPC < 3)			(3 ≤ PCPC)				
	n = 23			n = 11				
**Age in months, median (IQR), n**	**24**	**(18–35.5)**	**23**	**15**	**(10–18)**	**11**	**0.423**	0.014*
Sex, Female, n (%)	14	(60.9)		4	(36.4)		0.230	0.274
GCS, median (IQR), n	15	(12–15)	23	13	(9.5–14.5)	11	0.238	0.165
Worst GCS 24 hours post 1st seizure	14	(10–14.5)	20	9	(7–10.5)	6	0.377	0.062
AST, IU/L, median (IQR), n	40	(36.5–54)	23	49	(38.5–68)	11	0.171	0.320
ALT, IU/L, median (IQR), n	15	(12.5–23.5)	23	17	(14.5–34)	11	0.190	0.268
LDH, IU/L, median (IQR), n	330	(313–370)	23	329	(306–364)	11	0.022	0.897
Creatinine, mg/dL, median (IQR), n	0.32	(0.26–0.39)	23	0.28	(0.27–0.31)	11	0.070	0.685
Platelet, x10^3^/μL, median (IQR), n	27.4	(21.6–33.6)	23	28.1	(17.2–37.4)	11	0.003	1.000
Blood glucose, mg/dL, median (IQR), n	214	(136–252)	23	186	(155–247.5)	11	0.003	0.985
Duration of seizure in min, median (IQR), n	40	(7.5–60)	23	50	(42–63)	11	0.196	0.258
Distribution of the lesion Hemisphere, n (%)	8	(34.8)		4	(36.4)		0.207	0.417
Bilateral Frontal, n (%)	6	(26.1)		1	(9.1)			
Others, n (%)	9	(39.1)		6	(54.5)			
Diffuse lesion including perirolandic area, n (%)	1	(4.3)		2	(18.1)		0.183	0.239
Basal ganglia or thalamus lesion, n (%)	6	(26.1)		7	(63.6)		0.361	0.060
Patients with flu infection, n (%)	7	(30.4)		1	(9.0)		0.235	0.227
**Tada score, median (IQR), n**	**3**	**(2–5)**	**23**	**5**	**(3.5–6)**	**11**	**0.344**	0.045*
Yokochi score, median (IQR), n	3	(0–4.5)	23	3	(2–6)	11	0.058	0.737
Patients with biphasic clinical course, n (%)	16	(70.0)		6	(54.5)		0.542	0.542
**Duration from 1st seizure to 2nd phase (Time**^**1st-2nd**^**) in hours, median (IQR), n**	**107.5**	**(87.5–117)**	**16**	**76**	**(64–88)**	**6**	**0.582**	0.005*
Therapeutic options Steroid, n (%)	11	(47.8)		4	(36.3)		0.061	0.715
IVIG, n (%)	5	(21.7)		3	(27.3)		1.000	1.000
Cooling, n (%)	14	(60.9)		10	(90.9)		0.308	0.113

*p < 0.05; GCS, Glasgow Coma Scale; flu, Influenza virus.

**Table 2:** Comparison of clinical features between favourable and unfavourable outcome groups –continued.

	Favourable outcome			Unfavourable outcome			Effect size	p value
	(PCPC < 3)			(3 ≤ PCPC)				
	n = 23			n = 11				
Treatment option group, n (%)							0.406	0.077
Early-Hypo group	3	(13.0)		5	(45.5)			
Late-Hypo group	11	(47.8)		5	(45.5)			
Non-Hypo group	9	(39.1)		1	(9.1)			
Duration from 1st seizure, median (IQR), n								
to cooling initiation (Time_1st-cooling_)	94	(96.5–116	14	52.5	(26–91.5)	10	0.329	0.107
to 35 degrees Celsius (Time_1st-35 °C_)	97.5	(71–117.5)	14	54.5	(29–93.5)	10	0.335	0.101
to 34 degrees Celsius (Time_1st-34 °C_)	98.5	(25–118)	14	60	(38–94.5)	10	0.257	0.208
Duration from 2nd phase, median (IQR), n								
to cooling initiation (Time_2nd-cooling_)	9	(5.3–12)	11	10	(6.5–13)	5	0.114	0.650
to 35 degrees Celsius (Time_2nd-35 °C_)	10.5	(7–14.8)	11	11.5	(7–16)	5	0.085	0.733
to 34 degrees Celsius (Time_2nd-34 °C_)	11	(8–17.3)	11	14	(13–18)	5	0.184	0.461
Associated events								
Hypotension, n (%)	7	(30.4)		3	(27.2)		0.032	1.000
**Catecholamine use, n (%)**	**8**	**(34.8)**		**9**	**(81.8)**		**0.440**	0.026*
Pneumonia, n (%)	3	(13.0)		4	(36.4)		0.270	0.178
**Thrombocytopenia, n (%)†**	**9**	**(39.1)**		**9**	**(81.8)**		**0.440**	0.003*
**Minimal platelet x10**^**3**^**/μL, median (IQR), n**^**‡**^	**18.2**	**(12.3–24.3)**	**23**	**7.1**	**(4.9–12.0)**	**11**	**0.455**	0.007*
Minimal platelet excluding cooling period, x10^3^/μL, median (IQR), n^§^	19.5	(15.3–24.4)	23	18.2	(12.3–24.1)	11	0.031	1.000
Coagulation disorder, n (%)	16	(69.6)		10	(90.9)		0.235	0.227
Arrhythmia, n (%)	0	(0)		0	(0)		—	—
Hypokalaemia <3.5 mEq/L, n (%)	15	(65.2)		10	(90.9)		0.272	0.214
Dexmedetomidine use during cooling	5	(21.7)		3	(27.3)		0.061	1.000

*p < 0.05, ^†^Platelets <150 × 10^3^/μL, ^‡^Minimal platelet count within 10 days after 1^st^ seizure, ^§^Minimal platelet count within 10 days excluding cooling period, ^¶^PT > 12 s, PT INR > 1.2 s, or APTT > 45 s.

### Determinants of outcomes for the overall AED population

The multivariate logistic regression analysis revealed that the presence of basal ganglia/thalamus lesions on the MRI and the Tada score were independent variables associated with unfavourable outcomes (*p* = 0.027 and 0.032, respectively, Table 3).

**Table 3:** Multivariate logistic analysis for all patients (n = 34).

	Odds Ratio for unfavourable outcome	(95% Confidence Interval)	*p* value
Basal ganglia or thalamus lesion	**8.036**	**(1.267–50.965)**	0.027*
Tada score	**1.747**	**(1.048–2.912)**	0.032*

Unfavourable outcome: PCPC > 2, ^*^p < 0.05.

### Subgroup analysis of the timing of therapeutic hypothermia and second phase

**Figure 2:**
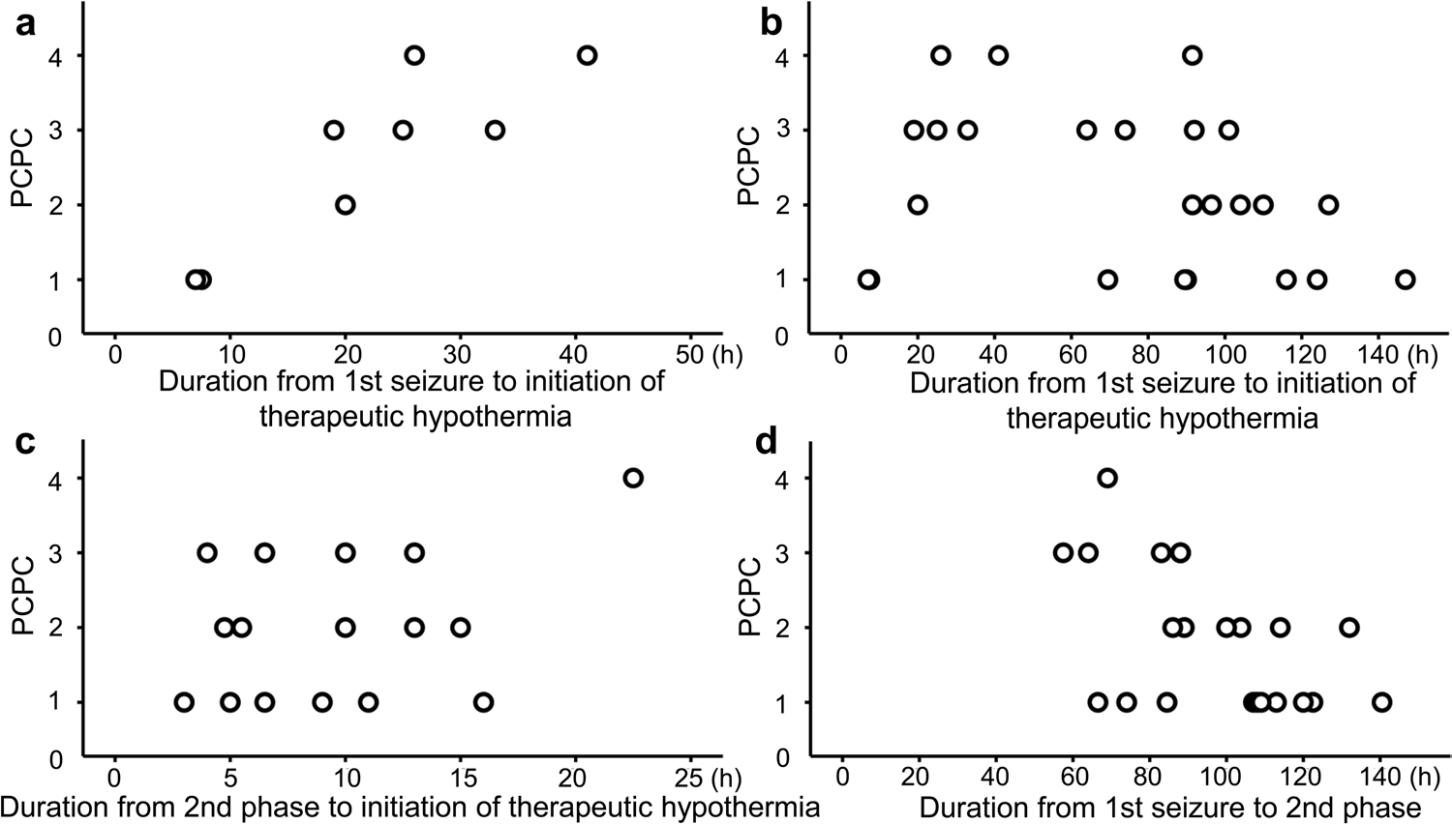
The relationships between outcomes and timing of therapeutic hypothermia **(a**–**c)** or the timing of the second phase **(d)**. **(a)** The relationship between outcomes represented by the PCPC score after 1 year and the Time_1st-cooling_ in the Early-Hypo group. Outcomes were dependent on the Time_1st-cooling_ (n = 8, *r* = 0.875, *p* = 0.004, Spearman’s rank correlation coefficient). The correlation analysis reached the statistical significance after adjustment by the Benjamini–Hochberg procedure to correct the multiple comparisons, using a false discovery rate of 0.05. **(b)** The relationship between the PCPC score after 1 year and the Time_1st-cooling_ in overall AED population with therapeutic hypothermia: the Early-Hypo and Late-Hypo groups. There was no association between the outcomes and the Time_1st-cooling_ (n = 24, *r* = 0.270, *p* = 0.270, Spearman’s rank correlation coefficient). **(c)** The relationship between the PCPC score after 1 year and the duration from initiation of the second phase to initiation of therapeutic hypothermia (Time_2nd-cooling_) in the Late-Hypo group. There was no association between the outcomes and the Time_2nd-cooling_ (n = 16, *r* = 0.208, *p* = 0.440, Spearman’s rank correlation coefficient). **(d)** The relationship between the PCPC score after 1 year and the duration from first seizure to initiation of the second phase (Time_1st-2nd_) in patients with AED with the second phase. No patients with Time_1st-2nd_ > 90 h had unfavourable outcome (3 ≤ PCPC). The outcomes were dependent on the Time_1st-2nd_ (n = 22, *r* = −0.495, *p* = 0.019, Spearman’s rank correlation coefficient). The correlation analysis reached the statistical significance after statistical significance was adjusted by the Benjamini–Hochberg procedure to correct multiple comparisons, using a false discovery rate of 0.05. AED, acute encephalopathy with reduced subcortical diffusion; PCPC, Paediatric Performance Category Scale; Time_1st-cooling_, duration from the first seizure to initiation of therapeutic hypothermia.

A strong association was found between the worse outcomes and the Time_1st-cooling_ (*p* = 0.004, Fig. 2a), and the duration from the first seizure to the time when the body temperature reached 35 **°**C (Time_1st-35°C_) (*p* = 0.002, Supplementary Fig. S10a) or 34 **°**C (Time_1st-34°C_) (*p* = 0.004, Supplementary Fig. S10b) in the Early-Hypo group. This association was statistically significant after adjustment by the Benjamini–Hochberg procedure to correct for multiple comparisons, using a false discovery rate of 0.05. There was no association between the outcomes and the Time_1st-cooling_ in the overall AED population with therapeutic hypothermia (Early-Hypo plus Late-Hypo group) (*p* = 0.270, Fig. 2b: Supplementary Fig. S12). Additionally, no association was observed between the outcomes and the Time_2nd-cooling_ (*p* = 0.440, Fig. 2c), and the duration between the first seizure to the time when the body temperature reached 35 °C (Time_2nd-35 °C_, *p* = 0.665, Spearman’s rank correlation coefficient) or 34 **°**C (Time_2nd-34 °C_, *p* = 0.366, Spearman’s rank correlation coefficient) in the Late-Hypo group.

In patients with AED with second phase, a moderate association was found between the outcomes and the Time_1st-2nd_ (*p* = 0.019, Fig. 2d). This association was statistically significant after adjustment by the Benjamini–Hochberg procedure to correct for multiple comparisons, using a false discovery rate of 0.05. No patients had any unfavourable outcomes with Time_1st-2nd_ of more than 90 h (Paediatric Performance Category Scale [PCPC] score > 2). There was a significant association between the Time_1st-2nd_ and the Time_1st-cooling_ in the Late-Hypo group (*p* < 0.001, Supplementary Fig. S11). This association was statistically significant after adjustment by the Benjamini–Hochberg procedure to correct for multiple comparisons, using a false discovery rate of 0.05. The cut-off value determined from the receiver operating characteristic (ROC) curve of Time_1st-cooling_ in the Early-Hypo group and the Time_1st-2nd_ in patients with AED with second phase to predict the unfavourable outcomes was 25 h (sensitivity, 100.0%; specificity, 80.0%; area under the curve [AUC], 0.933) and 83.75 h (sensitivity, 66.7%; specificity, 87.5%; AUC, 0.885; Supplementary Fig. S13), respectively.

## Discussion

This retrospective investigation of 34 patients with AED admitted to our institution over 14 years revealed three findings. First, patients with more severe consciousness disturbance between 12 and 24 h after the first seizure, had therapeutic hypothermia initiated before the second phase, and the most patients with therapeutic hypothermia received catecholamine or thrombocytopenia. Second, the presence of basal ganglia/thalamus lesions observed by the MRI and the Tada score were the determinants of poor outcomes in the multivariate analysis. Third, despite interpreting from the data from the small population and with many confounding factors existed, there was a strong association between the worse outcomes and the Time_1st-cooling_ in the Early-Hypo group, while no association was observed between the outcomes and the Time_1st-cooling_ or the Time_2nd-cooling_ in the overall AED population with therapeutic hypothermia. There was a moderate negative association between the worse outcomes and the Time_1st-2nd_.

### Therapeutic hypothermia use for AED population

In our study, the patients with more severe consciousness disturbance between 12 and 24 h after the first seizure tended to have therapeutic hypothermia initiated before the second phase. Meanwhile, as the worst GCS at 24 h after the first seizure in the Late-Hypo group was significantly lower and the number of patients with biphasic clinical course in the Late-Hypo group was significantly larger than those in the Non-Hypo group, we assumed that the attending physicians attempted to avoid therapeutic hypothermia when a patient had a milder clinical course while being unable to justify the continued use of sedations and intratracheal intubation. While therapeutic hypothermia may be a feasible therapy for this patient group considering the possibility that some patients with AED have severe brain oedema like our case with brain herniation (Supplementary Table S2a–c, Patient No. 1: Supplementary Fig. S6), therapeutic hypothermia could be associated with adverse effects and unfavourable outcomes when it is not managed properly. Hypotension is associated with therapeutic hypothermia in paediatric patients with head injury or post-cardiac arrest^14,15^ and thrombocytopenia has also been reported being associated with therapeutic hypothermia in adult head injury patients and infants with hypoxic-ischaemic encephalopathy^16,17^. Additionally, hypotension and low cerebral perfusion pressure were associated with unfavourable outcomes in randomised control in children with severe traumatic brain injury^15^. Further, therapeutic hypothermia was found to be associated with hypotension, catecholamine use, thrombocytopenia, and hypokalaemia in our current study (Supplementary Table S1b) particularly considering that there were no patients with hypotension, catecholamine use, thrombocytopenia, or hypokalaemia, except for the two patients with thrombocytopenia (Supplementary Table S2b,e, Patient No. 16 and 33) and two patients with hypokalaemia (Patient No. 10 and 15) after the first seizure. The previous retrospective study demonstrated that the platelet count was reduced after the first seizure and the minimal platelet count within the first 4 days was higher in the favourable than in the unfavourable outcome group among 10 patients with AED without therapeutic hypothermia^18^. In our study, the minimal platelet count within the first 10 days after the first seizure was significantly lower in the unfavourable than in the favourable group (*p* = 0.007, Table 2). However, no significant difference was found in the platelet count after the first seizure or the minimal platelet count within 10 days after the first seizure excluding the cooling period between the favourable and unfavourable outcome groups. Preventing hypotension during therapeutic hypothermia is critical with the appropriate dose of sedatives, fluids, and inotropes. Dexmedetomidine use, combined with therapeutic hypothermia, was reportedly associated with neurotoxicity in a preclinical study, presumably due to the reduction of dexmedetomidine clearance leading to bradycardia, hypotension, and cardiac arrest^19,20^, while a successful use of dexmedetomidine with reduced dose during therapeutic hypothermia has been reported in infants with hypoxic-ischaemic encephalopathy^22^. In this study, dexmedetomidine was used in eight patients during therapeutic hypothermia without increasing the adverse events, such as prolonged hypotension or cardiac arrest even though the titration of the dosage required was 0.2–0.9 μg/kg/h (Supplementary Tables S1b and S2b,e). However, the appropriate dose of sedations including dexmedetomidine with therapeutic hypothermia in paediatric patients and the effect of the long-term use of these sedations need to be further clarified.

### Determinants of outcomes for the overall AED population

The association between the basal ganglia lesions and poor outcomes has already been reported in univariate analysis^7^, and the multivariate analysis result of our current study is consistent with those findings (Table 3). Diffuse lesions with injury around perirolandic regions, prolonged seizures at onset, coma, or involuntary movements have been associated with poor outcomes in other retrospective studies^1,2,7^. All three patients with diffuse lesions with injury around the perirolandic regions observed on the MRI in our study had basal ganglia/thalamus lesions, coinciding with a previous report that patients with AED with diffuse or basal ganglia lesions represent a severe phenotype of acute encephalopathy^2,7^. Diffuse lesions without injury around the perirolandic regions on DWI were observed in patients after the first 3 days from the onset and a milder phenotype of patients with AED^2^, meanwhile, patients with diffuse lesions with injury around the perirolandic regions on DWI, were reported to be of a severe phenotype of AED, first showing frontal and occipital lesions within the first 3days from the onset and later exhibiting diffuse lesions on DWI. However, two patients were observed with diffuse lesions without injury around the perirolandic regions on DWI within the first 3 days from onset (Supplementary Table S2a,d, Patient No. 4 and 26) and no patients exhibited lesions on DWI spreading on the second MRI after the first abnormal MRI. This difference might be due to the previous report being based on a small number of patients with AED (nine patients). Moreover, we did not have a patient with bilateral diffuse lesions with injury around the bilateral perirolandic regions in the study population. According to the results of the univariate analysis in our study, no significant difference was observed in the duration of the first seizure and the GCS score, at 12–24 h after the first seizure between the favourable and the unfavourable outcome groups. For the duration of the first seizure, this coincides with the results of the report by Lee *et al*., which included 17 patients with therapeutic hypothermia out of 20 patients with AED^7^, while Hayashi *et al*. reported that prolonged seizures at onset were a predictor for poor outcomes in 33 patients with AED, none of whom were cooled^1^. Therapeutic hypothermia may have altered the outcomes; however, further studies are warranted for clarification.

### Time-dependent association between the initiation of therapeutic hypothermia and the outcome

The strong association between worse outcomes and the longer Time_1st-cooling_ in the Early-Hypo group is consistent with the results of our previous study in children with various types of acute encephalopathy/encephalitis, in terms of the efficacy of early initiation of therapeutic hypothermia^21^, although the positive effect of therapeutic hypothermia compared to normothermia treatment is difficult to be determined in this study. Indeed, the study population was small and there were many confounding factors regarding the hypothermia initiation, such as the attending physicians’ judgement to apply therapeutic hypothermia. The cut-off value from the ROC curve of the Time_1st-cooling_ in the Early-Hypo group to predict unfavourable outcomes was 25 h; however, this was determined from the data consisting of a small number of the population. Additionally, when the therapeutic hypothermia had been sufficient in preventing the appearance of lesions with subcortical reduced ADC, we would have had patients without abnormal findings on MRI after the therapeutic hypothermia; however, we did not have any such patient without abnormal findings on MRI taken after therapeutic hypothermia was completed (Supplementary Fig. S7). This highlights the need for developing adjunctive therapies to improve the outcomes of patients with AED. The efficacy of therapeutic hypothermia has been proven for neonatal hypoxic encephalopathy and adult post-cardiac arrest syndrome^8–11^. The cerebral metabolic rate reduction, enhancing glucose utilisation, inhibiting free radical production, and the inhibition of inflammatory responses and apoptotic pathways have been demonstrated, as the mechanisms for therapeutic hypothermia. However, the aforementioned have yet to be fully elucidated. A preclinical study using a rat model of traumatic brain injury revealed that therapeutic hypothermia downregulated connexin 43 expression and upregulated the levels of glutamate transporter 1 (GLT-1) in the hippocampus^23^. Connexin 43 forms the major component protein in astrocytic gap junction and is thought to participate in the pathophysiological processes of brain damage^24^. Glutamate is a major excitatory neurotransmitter in the central nervous system; however, high concentration of glutamate in the extracellular space caused by status epilepticus, hypoxic-ischaemic insult, or traumatic brain injury is neurotoxic. GLT-1, predominantly expressed in astrocytes, provides the majority of glutamate clearance capacity by transporting it into the intracellular space^25^. The pathological findings of a patient with acute encephalopathy with biphasic seizures and late reduced diffusion at 6 days after the second phase onset, revealed a reduction of the myelinated axons and an increased number of gemistocytic astrocytes at the corticomedullary junction, where bright tree appearance was observed^26^. Axonal degeneration is postulated to stem from an increased influx of Ca^2+^ into the axons leading to impaired axonal mitochondrial metabolism, glutamate-mediated oligodendrocyte damage, decreased production of N-acetylaspartate by axonal mitochondria, and glutamate excitotoxicity. The astrocytic gap junctions and glutamate uptake in astrocytes are speculated to be already disrupted to some point in these gemistocytic astrocytes. This might explain the association between the earlier therapeutic hypothermia initiation and the better outcomes only in the Early-Hypo group.

In contrast, delayed and prolonged therapeutic hypothermia are reported to be associated with white matter apoptosis in preclinical reports^27,28^. Glutamate release from astrocytes to the adjacent neurons triggered by the Ca^2+^ release from the astrocytes’ intracellular stores following extracellular glutamate stimulation was demonstrated^29^. The effect of delayed and prolonged therapeutic hypothermia may oppositely contribute to releasing glutamate from the astrocytes through upregulation of GLT-1^23,30^. This may explain why the relationship between the earlier therapeutic hypothermia initiation and the better outcomes was not observed in the Late-Hypo group in this study. Therapeutic hypothermia after the second phase may not be as effective compared to that initiated before the second phase. The role of astrocytes should be further clarified in the time course in patients with AED to maximise the benefit of the available treatment options, including therapeutic hypothermia as the astrocytes are known to have dual functions of glutamate uptake and release^31^. Diagnosis of AED before the second phase is challenging because of the absence of abnormal neuroradiological, characteristic laboratory, or clinical findings. The Yokochi score is another AED prediction score, similar to the Tada score, that was proposed to recognise this disease before the second phase; however, its positive predictive value was 47% and the initiation of therapeutic hypothermia according to this score has yet to be justified^32^. Thus, in the most AED cases in other facilities, therapeutic hypothermia could be initiated after the second phase, similar to our institution. Considering that the Japanese guideline for acute encephalopathy in childhood do not recommend therapeutic hypothermia for patients with acute encephalopathy^12^, the relationship between early therapeutic hypothermia and superior outcomes in the Early-Hypo group may justify future investigations on whether early induction of therapeutic hypothermia in patients after a febrile seizure is effective with a more refined AED prediction score.

The initiation of therapeutic hypothermia inevitably implies the initiation of adequate sedation, necessitating the continuous infusion of midazolam or thiamylal sodium. Thus, we cannot exclude the possibility that early initiation of these medications suppressed the second phase and played a role in alleviating outcomes instead of the direct effects of therapeutic hypothermia for brain damage. However, this does not explain the time-dependent effects of therapeutic hypothermia in the Early-Hypo group, as all patients in this group had equally suppressed seizures in the second phase.

### Worse outcomes and the earlier second phase arrival

The patients with unfavourable outcomes had a shorter Time_1st-2nd_ compared to those with favourable outcomes in the univariate analysis. This result coincided with that of a preclinical study on newborn piglets reporting that more severe hypoxia-ischaemia insults were associated with a shorter latent-phase, worse secondary energy failure, and more cortical-grey-matter neuronal death^33^. This association between the Time_1st-2nd_ and the outcomes may also explain why the relationship between the earlier therapeutic hypothermia initiation and the better outcomes was not observed in the Late-Hypo group in this study. Previous clinical studies with AED^1,7^ did not examine this relationship between the outcomes and the Time_1st-2nd_ in patients with AED. We were unable to clarify which factors were attributed to the early arrival of the second phase in patients with AED as no variables except the presence of therapeutic hypothermia treatment, catecholamine use, or thrombocytopenia correlated with the Time_1st-2nd_.

### Limitations of this study

This study was a retrospective observational study with a small number of patients with AED in a single centre. One limitation was that this study did not intend to evaluate the efficacy of therapeutic hypothermia, as the decision to whether to cool patients or not was based on the attending physicians’ judgement when the patients had a GCS score > 8. All patients with AED that received therapeutic hypothermia were cooled to 34 °C for 72–84 h according to the protocol in our facility, occluding us from examining the difference in efficacy between the temperature management at 34°C or 36 °C. Future prospective randomised trials are warranted to directly compare the neurodevelopment scores, such as the Wechsler Preschool and the Primary Scale of Intelligence-fourth edition or the Wechsler Intelligence Scale for Children (WISC) -fifth edition, which are considered to be more sensitive than the PCPC scale when examining the difference in efficacy between the temperature management at 34 °C or 36 °C in patients with AED. Second, all data were retrospectively collected from medical records and it is unlikely that every involuntary movement before the second phase was recorded for each patient, which has been reported to be associated with poor outcomes^7^. Thus, we did not examine episodes of involuntary movement. Prospective studies with more patients are required to examine the association of various clinical symptoms and features with outcomes. Third, we did not routinely check for the anti-N-methyl-D-aspartate receptor (NMDA) and the anti-myelin oligodendrocyte glycoprotein (MOG) antibodies, and we did not conduct a polymerase chain reaction (PCR) test for microorganisms in the CSF in each patient. Therefore, we were unable to examine the possible contribution of these mechanisms to AED and the outcome of patients with AED.

This study revealed that the presence of basal ganglia/thalamus lesions on the MRI and the Tada score were the determinants of poor outcomes in patients with AED. Further investigation is needed to examine the efficacy of therapeutic hypothermia in this population while considering the timing of the therapeutic hypothermia initiation and the second phase.

## Methods

### Study population

After excluding patients diagnosed with acute disseminated encephalomyelitis (ADEM), multiple sclerosis (MS), autoimmune encephalitis, traumatic encephalopathy, mild encephalitis/encephalopathy with a reversible splenial lesion^34^, posterior reversible encephalopathy syndrome, and hepatic encephalopathy, we had a total of 50 children aged between 1 month and 14 years, who developed or were suspected of having acute encephalopathy. They were admitted in our hospital between February 2004 and August 2018. Especially, 37 patients received therapeutic hypothermia of whom 26 were diagnosed as having AED. Thirteen patients were not provided therapeutic hypothermia, of whom 10 were diagnosed as having AED (Supplementary Fig. S1). After excluding 2 AED patients with neurodevelopment delay before onset from the total of 36 AED patients, 34 AED patients were retrospectively enrolled in this study (Fig. 1, Supplemental Fig. S1). The diagnosis of AED was defined as (1) impaired consciousness with or without other neurological symptoms, (2) a febrile seizure as the first neurological symptom, and (3) reduced ADC in subcortical white matter/bright tree appearance in DWI within 2 weeks after the first seizure regardless of the presence or absence of the second phase. The CSF was examined in 28 out of 34 patients with AED (Supplementary Table S2c,f). A second CSF examination was done in two patients (Patient No. 3 and 4) other than the patient with septic meningitis (Patient No. 18). We did not routinely check for the anti-NMDA receptor or the anti-MOG antibodies in the CSF in each patient as all patients with AED did not have neuroimaging findings of ADEM or MS, such as multiple high-intensity lesions in ADC suggesting demyelination defined by the criteria of the International Pediatric Multiple Sclerosis Group in 2013, or typical clinical features for autoimmune encephalitis, such as orofacial or limb dyskinesias, tremor, choreoathetosis, or dysautonomia. Preceding infection was identified in 16 patients, of whom eight had influenza virus, followed by human herpesvirus 6 (n = 2), varicella-zoster virus (n = 2), adenovirus, coxsackievirus A9, rotavirus, and streptococcus pneumoniae meningitis. Preceding infections were confirmed with at least one positive result of viral culture, antigen test, reverse transcription PCR, or significantly raised titres in paired serum samples. Three other patients were clinically diagnosed with exanthem subitem, and another patient was diagnosed with Kawasaki disease. No pathogens were identified in 14 patients (Supplementary Table S2a,d). Although we confirmed the absence of herpes simplex virus infection by using CSF herpes simplex virus (HSV) DNA or serum anti-HSV IgG in each patient, we also did not routinely examine the CSF viral culture or conduct the PCR test for other viruses in each patient.

We defined the first seizure of the second phase as its initiation, as the initiation of consciousness disturbances is typically challenging to be detected, especially in young children and during night, even though consciousness disturbances are often accompanied by precede repetitive seizures. Additionally, the timing of seizures was recorded precisely in all patients compared to the time of the initiation of consciousness disturbances, providing more accurate and objective data considering the nature of this retrospective study. All patients visited our or referring hospitals immediately after the first seizure, apart from one patient (Supplementary Table S2d–f, Patient No. 19) in the Non-Hypo group, who was transferred to our hospital after the initiation of the second phase. Hence, the results of the blood test on arrival were used and the timing and duration of the first seizure were speculated according to her parents’ description. One patient cooled before the second phase was included in our previous study for patients with various types of acute encephalopathy/encephalitis in children^21^. Nineteen patients were included in the previous study for prediction of acute encephalopathy with biphasic seizures and late reduced diffusion in patients with febrile status epilepticus^32^. This study was approved by the institutional review board of St Mary’s Hospital, Fukuoka, Japan (IRB number: 18–1110). All methods were performed following the relevant guidelines and regulations. Informed consent was obtained from all participants and/or their legal guardians.

### Treatment of encephalopathy

Therapeutic hypothermia has been used for managing brain oedema in patients with AED at several tertiary centres in Japan^7,21,35^ despite the lack of randomised controlled trials in such patients. The 2016 Japanese practice guideline for acute encephalopathy in childhood^12^ does not recommend this treatment. Conversely, the American Heart Association paediatric advanced life support guidelines recommend targeted temperature management as the standard of treatment care in managing paediatric post-cardiac arrest syndrome even though there is no moderate/high-quality evidence^36^. Therefore, we have been providing therapeutic hypothermia for this patient group throughout the study period following our institutional protocol for managing patients with consciousness disturbance (Supplementary Data). According to the protocol, therapeutic hypothermia should be indicated for patients with continuous consciousness disturbance and haemodynamically stable condition. The contraindication is patients with head trauma or severe coagulation disorders. When a patient continuously had a GCS score <9, therapeutic hypothermia was applied. When a patient had a GCS score> 8, the decision to apply therapeutic hypothermia or not was taken according to the attending physicians’ judgement on the severity of neurological symptoms for that patient. All patients with therapeutic hypothermia were intubated, mechanically ventilated, and cooled with mattresses with temperature-adjustable water circulating over the ventral and dorsal trunks following our institute’s protocol. The core temperature was monitored using more than two of the oesophageal, nasopharyngeal, rectal, or urinary bladder temperature probes. The target temperature was 34 °C and the cooling duration was 72 h. During cooling, continuous infusion of thiamylal sodium (up to 3 mg/kg/h) or midazolam (up to 0.6 mg/kg/h) and fentanyl (1–3 μg/kg/h) were administered. Continuous midazolam infusion was administered in two patients (Supplementary Table S2a–c, Patients No. 1 and 2) and continuous thyamiral infusion was administered for the remaining patients combined with fentanyl during therapeutic hypothermia. In addition to these medications, dexmedetomidine, a selective α2-adrenoreceptor agonist, was administered in eight patients at 0.5 μg/kg/h, which was increased to 1.0 μg/kg/h for the first hour in three patients, with the titillation at 0.2–0.9 μg/kg/h according to the vital signs and the level of sedation during the period from the initiation of therapeutic hypothermia until the day or a few days after extubation (Supplementary Table S2b, Patients No. 9–18). Intravenous methylprednisolone pulse (30 mg/kg/dose × 3 days) therapy or immunoglobulin (1 g/kg/dose) were used according to the attendant physicians’ judgement before February 2017, as these treatments had been suggested as the adjunctive therapies to conventional respiratory, haemodynamic management, and seizure control in the Japanese guidelines for influenza encephalopathy despite the lack of evidence from randomised control studies. However, we refrained using these two therapies for patients with AED since March 2017 (Supplementary Table S2d, Patients No. 23–25 and 27–34) except for one patient with Kawasaki disease (Supplementary Table S2d, Patient No. 26), as the 2016 Japanese practice guideline for acute encephalopathy in childhood suggested that these therapies may be effective only for cases with encephalopathy with hypercytokinemia, such as acute necrotising encephalopathy. Dexmedetomidine use was not mentioned in our institute’s protocol, but this was administered routinely to patients with AED with therapeutic hypothermia from July 2011 to April 2014 in our facility to prevent shivering. Occasional intravenous vecronium or pancuronium was administered for uncontrollable shivering. Patients were rewarmed 0.05–0.1 °C per h. All patients were classified in one of the following three groups (Fig. 1): 1) Patients who were started on therapeutic hypothermia by an attending physician before the second phase due to a worsening level of consciousness or continuously impaired consciousness (Early-Hypo group). Reduced ADC in the subcortical white matter on brain MRI was confirmed within 2 weeks of initiating therapeutic hypothermia in all patients in the Early-Hypo group except for one patient, whose MRI already showed reduced ADC in the diffuse white matter lesions immediately after the first seizure (Supplementary Table S2a–c, Patient No. 14); 2) patients who started therapeutic hypothermia after the initiation of the second phase (Late-Hypo group); 3) patients who were not provided therapeutic hypothermia (Non-Hypo group). The reason for not providing therapeutic hypothermia was due to mildness of clinical neurological presentation (n = 6) or absence of the second phase (n = 4). All the patients in the Non-Hypo group were not intubated, mechanically ventilated, administered with continuous infusion of sedation, or maintained the body temperature with the cooling device. The timing of the second MR imaging was delayed in the Early-Hypo group until the end of therapeutic hypothermia due to the difficulties in maintaining body temperature and vital signs during the transport to the MRI room, and the MR image acquisition in our facility, thus, delaying the timing of obtaining the abnormal MRI (Supplementary Table S1b, Supplementary Fig. S8).

### Data collection

Clinical variables were collected for each patient including age; sex; associated infections; first seizure duration; GCS scores between 12 and 24 h after the first seizure; the worst GCS score after 24 h from the first seizure in the Late-hypo and Non-Hypo groups excluding the ictal and postictal states, and at 3 h after intravenous administration of antiepileptic medication; treatment option (Early-Hypo, Late-Hypo, or Non-Hypo group); Time_1st-2nd_ in the late-Hypo and non-Hypo groups when the second phase existed; Time_1st-cooling_, Time_1st-35 °C_ and Time_1st-34 °C_ in the Early-Hypo and Late-Hypo groups; Time_2nd-cooling_, Time_2nd-35 °C_ and Time_2nd-34 °C_ in the Late-Hypo group; distribution of brain lesions on the MRI (hemisphere, bilateral frontal lobe, or others); days when the first MR image was taken from the first seizure; days when reduced subcortical ADC confirmed on the MRI from the first seizure; presence of diffuse lesions with injury around the perirolandic regions on the MRI at least in a hemisphere; presence of basal ganglia/thalamus lesions on MRI; potential serum markers of tissue damage such as aspartate aminotransferase (AST), alanine aminotransferase (ALT), and lactate dehydrogenase after the first seizure; blood urea nitrogen, Crea, Glu, platelet count after the first seizure; cell count and protein level in the CSF and the timing of the CSF examination; the Tada and the Yokochi scores after the first seizure^13,33^; preceding infections; additional treatments, such as intravenous methylprednisolone pulse (30 mg/kg/dose × 3 days) therapy, or immunoglobulin (1 g/kg/dose); the presence and the dosage of dexmedetomidine use during therapeutic hypothermia, and outcomes. The Tada score is a predicting score for acute encephalopathy with biphasic seizures and late reduced diffusion among patients with febrile seizure in the acute phase and consisted of consciousness level, duration of convulsions, enforcement of mechanical intubation, AST, Glu, and Crea^13^. The Yokochi score is another similar predicting score for acute encephalopathy with biphasic seizures and late reduced diffusion^33^. Events of hypotension (defined as systolic blood pressures below average values -2SD for patients’ age following the Task Force on Blood Pressure Control in Children^37^) regardless of the catecholamine use, catecholamine use requirement, pneumonia, thrombocytopenia (<150,000/μL), coagulation disorders (prothrombin time >12 s, international normalised ratio of prothrombin time >1.2 s or activated partial thromboplastin time >45 s), arrhythmias (excluding bradycardia), and hypokalaemia (<3.5 mEq/L) were also recorded. Regarding thrombocytopenia, the minimal platelet count within 10 days after the first seizure and minimal platelet count within 10 days excluding the cooling period were recorded.

Additionally, we collected the data regarding the presence of abnormal MRI findings in patients with acute encephalopathy types other than AED, who were performed therapeutic hypothermia during this study period, to check for those in whom therapeutic hypothermia could have prevented the appearance of abnormal MRI findings (Supplementary Fig. S1).

### Outcome assessment

The neurological outcomes at 12 months from disease onset were assessed using the PCPC score^38^ from medical records by two paediatricians for each patient including the results of the neurodevelopment evaluation by the Kyoto Scale of Psychological Development 2001, the Tanaka-Binet Intelligence Test, or the WISC-III or IV for each patient, which were obtained by clinical psychologists who were unaware of the treatment assignment. The two paediatricians reached to agreement after discussion for patients whose PCPC scores differed between them. For patients who did not have any clinical records at 12 months from onset, assessments were made using clinical records from 3 months before or after the point. The PCPC scores of 1–6 represent normal, mild, moderate, and severe disabilities, coma or vegetative state, and death, respectively^38^. The PCPC scores of 1 or 2 and those of 3–6 were classified as favourable and unfavourable outcomes, respectively.

### Statistical analysis

SPSS ver.25 (IBM Corp., Armonk, NY, USA) was used for all statistical analyses. First, to examine the differences between the favourable and unfavourable outcome groups, clinical variables and treatment options were examined using the Mann-Whitney *U* test (two-tailed) for numerous variables or the Fisher’s exact test (two-tailed) for numerous variables with effect sizes of *r* and *phi* for 2 × 2 tables or the Cramer’s *V* for those other than 2 × 2 tables. The statistically significant level was set at *p* < 0.05.

Second, we performed the multivariate logistic regression analysis for worse outcomes within the patients with AED. In addition to the treatment option (therapeutic hypothermia or not), the variables that showed *p*-values equal to or less than 0.06 in the univariate intergroup analysis except for The Time_1st-2nd_, the worst GCS scores after 24 h from the first seizure, the presence of catecholamine use, and thrombocytopenia, were included in a multivariate logistic regression analysis with backwards stepwise elimination of non-significant variables. The Time_1st-2nd_ and the worst GCS scores after 24 h from the first seizure were excluded from the analysis, as these variables were not for all the patients, such as the Time_1st-2nd_ only for patients with AED with second phase, the later one only for the patients in the Late-Hypo group and Non-Hypo group (Supplementary Table S1a,b). To avoid interference by multicollinearity, each association between the two variables among age, Tada score, the presence of basal ganglia/thalamus lesions, treatment option (therapeutic hypothermia or not), catecholamine use and thrombocytopenia was examined. The Mann-Whitney *U* test (two-tailed) was used for the associations between the Tada score and other categorical variables and Fisher’s exact test (two-tailed) was used for the other associations. The statistical significance was adjusted by the Benjamini–Hochberg procedure to correct for multiple comparisons of 10 times, using a false discovery rate of 0.05. The statistical significant associations were found between therapeutic hypothermia and catecholamine use, therapeutic hypothermia and thrombocytopenia, catecholamine use and thrombocytopenia, and the Tada score and catecholamine use (*Phi* = 0.645, p < 0.001; *Phi* = 0.685, *p* < 0.001; *Phi* = 0.471, *p* = 0.015; *r* = 0.475, *p* = 0.008, respectively). Thus, the presence of catecholamine use and thrombocytopenia were also excluded from the multivariate logistic analysis.

Third, we performed subgroup analysis examining the relationships between outcomes in the Early-Hypo group and the Time_1st-cooling_, or the Time_1st-35 °C_, and the Time_1st-34 °C_; outcomes in patients with AED with therapeutic hypothermia and the Time_1st-cooling_; outcomes in the Late-Hypo group and the Time_2nd-cooling_; outcomes in patients with AED with second phase and the Time_1st-2nd_ using the Spearman’s rank correlation coefficient. The relationship between the Time_1st-cooling_ and the Time_1st-2nd_ in the Late-Hypo group was also examined to investigate the influence of second phase initiation on the timing of therapeutic hypothermia using the Pearson’s correlation coefficient as the two variables were normally distributed. Here, the statistical significance was adjusted by the Benjamini–Hochberg procedure to correct multiple comparisons of seven times, using a false discovery rate of 0.05. Additionally, the threshold of Time_1st-cooling_ in the Early-Hypo group and Time_1st-2nd_ in patients with AED with second phase were determined to predict unfavourable outcomes from the ROC curve.

### Data availability statement

All data generated or analysed during this study are available from the corresponding author on reasonable request.

## Supplementary information

Supplementary Information.

## References

[1] Hayashi N (2012). Prognostic factors in acute encephalopathy with reduced subcortical diffusion. Brain Dev..

[2] Okumura A (2009). Differences of clinical manifestations according to the patterns of brain lesions in acute encephalopathy with reduced diffusion in the bilateral hemispheres. Am. J. Neuroradiol.

[3] Takanashi J (2006). Diffusion MRI abnormalities after prolonged febrile seizures with encephalopathy. Neurology.

[4] Yadav S, Lawande M, Kulkarni S, Patkar D (2013). Acute encephalopathy with biphasic seizures and late reduced diffusion. J. Pediatr. Neurosci.

[5] Hoshino A (2012). Epidemiology of acute encephalopathy in Japan, with emphasis on the association of viruses and syndromes. Brain Dev..

[6] Mizuguchi M, Yamanouchi H, Ichiyama T, Shiomi M (2007). Acute encephalopathy associated with influenza and other viral infections. Acta Neurol. Scand. Suppl..

[7] Lee S (2016). Involuntary movements and coma as the prognostic marker for acute encephalopathy with biphasic seizures and late reduced diffusion. J. Neurol. Sci..

[8] Bernard SA (2002). Treatment of comatose survivors of out-of-hospital cardiac arrest with induced hypothermia. N. Engl. J. Med..

[9] The hypothermia after cardiac arrest study group (2002). Mild therapeutic hypothermia to improve the neurologic outcome after cardiac arrest. N. Engl. J. Med..

[10] Shankaran S (2005). Whole-body hypothermia for neonates with hypoxic–ischemic encephalopathy. N. Engl. J. Med.

[11] Jacobs, S. E. *et al*. Cooling for newborns with hypoxic ischaemic encephalopathy. *Cochrane database Syst. Rev*. CD003311 (2013). 10.1002/14651858.CD003311.pub3 PMC7003568 23440789

[12] Japanese Society of Cild Neurology. *Guideline for acute encephalopathy in childhood*. Chapter 3, 46–55 (Shindan To Chiryosha, 2016).

[13] Tada H (2015). Predictive score for early diagnosis of acute encephalopathy with biphasic seizures and late reduced diffusion (AESD). J. Neurol. Sci..

[14] Hutchison JS (2008). Hypothermia therapy after traumatic brain injury in children. N. Engl. J. Med.

[15] Hutchison JS, Frndova H, Guerguerian AM (2010). Hypothermia Perdiatric Head Injury Trial Investigators & Canadian Critical Care Trials Group. Impact of hypotension and low cerebral perfusion pressure on outcomes in children treated with hypothermia therapy following severe traumatic brain injury: a post hoc analysis of the hypothermia pediatric head injury trial. Dev. Neurosci..

[16] Metz C (1996). Moderate hypothermia in patients with severe head injury: cerebral and extracerebral effects. J. Neurosurg.

[17] Zhang W, Ma J, Danzeng Q, Tang Y, Lu M, Kang Y (2017). Safety of moderate hypothermia for perinatal hypoxic-ischemic encephalopathy: a meta-analysis. Pediatr. Neurol..

[18] Azuma J (2015). Prognostic factors for acute encephalopathy with bright tree appearance. Brain Dev..

[19] Ezzati M (2014). Pharmacokinetics of dexmedetomidine combined with therapeutic hypothermia in a piglet asphyxia model. Acta Anaesthesiol. Scand..

[20] Ezzati M (2017). Dexmedetomidine combined with therapeutic hypothermia is associated with cardiovascular instability and neurotoxicity in a piglet model of perinatal asphyxia. Dev. Neurosci..

[21] Kawano G (2011). Determinants of outcomes following acute child encephalopathy and encephalitis: pivotal effect of early and delayed cooling. Arch. Dis. Child..

[22] O’Mara K, Weiss M (2018). Dexmedetomidine for sedation of neonates with HIE undergoing therapeutic hypothermia: a single-center experience. Am. J. Perinatol. Reports.

[23] Li YH, Zhang CL, Zhang XY, Zhou HX, Meng LL (2015). Effects of mild induced hypothermia on hippocampal connexin 43 and glutamate transporter 1 expression following traumatic brain injury in rats. Mol. Med. Rep.

[24] Ohsumi A (2010). Temporal and spatial profile of phosphorylated connexin43 after traumatic brain injury in rats. J. Neurotrauma.

[25] Goodrich GS (2013). Ceftriaxone treatment after traumatic brain injury restores expression of the glutamate transporter, GLT-1, reduces regional gliosis, and reduces post-traumatic seizures in the rat. J. Neurotrauma.

[26] Takanashi JI (2018). Loss of myelinated axons and astrocytosis in an autopsy case of acute encephalopathy with biphasic seizures and late reduced diffusion. Brain Dev..

[27] Wang B (2016). White matter apoptosis is increased by delayed hypothermia and rewarming in a neonatal piglet model of hypoxic ischemic encephalopathy. Neuroscience.

[28] Sabir H, Scull-Brown E, Liu X, Thoresen M (2012). Immediate hypothermia is not neuroprotective after severe hypoxia-ischemia and is deleterious when delayed by 12 hours in neonatal rats. Stroke.

[29] Hamilton NB, Attwell D (2010). Do astrocytes really exocytose neurotransmitters?. Nat. Rev. Neurosci..

[30] Mitani A, Tanaka K (2003). Functional changes of glial glutamate transporter GLT-1 during ischemia: An *in vivo* study in the hippocampal CA1 of normal mice and mutant mice lacking GLT-1. J. Neurosci..

[31] Mahmoud S, Gharagozloo M, Simard C, Gris D (2019). Astrocytes maintain glutamate homeostasis in the CNS by controlling the balance between glutamate uptake and release. Cells.

[32] Yokochi T (2016). Prediction of acute encephalopathy with biphasic seizures and late reduced diffusion in patients with febrile status epilepticus. Brain Dev..

[33] Iwata O (2007). Therapeutic time window duration decreases with increasing severity of cerebral hypoxia-ischaemia under normothermia and delayed hypothermia in newborn piglets. Brain Res..

[34] Tada H (2004). Clinically mild encephalitis/encephalopathy with a reversible splenial lesion. Neurology.

[35] Murata S (2016). Targeted temperature management for acute encephalopathy in a Japanese secondary emergency medical care hospital. Brain Dev..

[36] Duff JP (2019). 2019 American Heart Association focused update on pediatric advanced life support: an update to the American Heart Association guidelines for cardiopulmonary resuscitation and emergency cardiovascular care. Circulation.

[37] Report of the second task force on blood pressure control in children—1987. task force on blood pressure control in children. National Heart, Lung, and Blood Institute, Bethesda, Maryland. *Pediatrics* **79**, 1–25 (1987). 3797155

[38] Fiser DH (1992). Assessing the outcome of pediatric intensive care. J. Pediatr..

