# Supplementary material for: Determinants of outcomes for acute encephalopathy with reduced subcortical diffusion
Source: Sci Rep. 2020 Jun 4;10:9134. doi: 10.1038/s41598-020-66167-7 (PMC7272444; doi:10.1038/s41598-020-66167-7)
Supplement: Supplementary file 1 — Supplementary Information. [file 41598_2020_66167_MOESM1_ESM.pdf]

Determinants of outcomes for acute encephalopathy with reduced subcortical diffusion

Kensuke Sakata, MD [1,a], Go Kawano, MD, PhD [1,a *], Masao Suda, MD [1], Takaoki Yokochi, MD, PhD [1], Yukako Yae, MD [2], Toru Imagi, MD [1] , Yukihiro Akita, MD, PhD [1], Keizo Ohbu, MD, PhD [1], Toyojiro Matsuishi, MD, PhD [1, 3]

[a~~1~~]These authors contributed equally to this work.

[1~~a~~]Department of Paediatrics, St Mary’s Hospital, 422 Tsubukuhonmachi, Kurume, Fukuoka 830-8543, Japan.

[2~~b~~] Department of Paediatrics, Kurume University Hospital, 67 Asahimachi, Kurume, Fukuoka 830-0011, Japan.

[3~~c~~]Research Centre for Children and Research Centre for Rett Syndrome, St Mary’s Hospital, 422 Tsubukuhonmachi, Kurume, Fukuoka 830-8543, Japan.

***Corresponding Author:**

Go Kawano, MD, PhD

Department of Paediatrics, St Mary’s Hospital, 422 Tsubukuhonmachi, Kurume, Fukuoka 830-8543, Japan.

Tel: +81-942-35-3322, FAX: +81-942-34-3115; E-mail: g-kawano@st-mary-med.or.jp

Supplemental Data: institutional protocol for therapeutic hypothermia

A. Indication

1. Children with prolonged consciousness disturbance
2. Encephalopathy after cardiac pulmonary arrest and acute encephalopathy associated with infectious diseases.

   Exclusion criteria: traumatic brain damage, intracranial haemorrhage, unstable vital signs, or severe coagulation disorders

B. Duration

1. Mild hypothermia 34℃ for 3 days (72 h)

   Rewarming could be postponed or slowed down with either the deterioration of neurological signs or the recurrence of brain oedema with the critically increased intracranial pressure. However, there was no evidence of prolonged hypothermia treatment.
2. Gradual rewarming (0.1℃ / h; to 36-36.5℃)

C. Induction Phase (Day 1) (In addition to systemic supportive care following Paediatric Advanced Life Support)

1. Start brain hypothermia treatment as soon as possible.
2. A head up position of 5-30° for severe brain swelling cases
3. Fluid resuscitation
4. Monitoring: ECG, Arterial blood pressure, Central venous pressure, SpO_2

   Consider intra cranial pressure (ICP) monitoring for severe brain swelling
5. Continuous EEG monitoring
6. Thermo-regulation: using the cooling machine with cooling blankets, as well as oesophagus and bladder temperature monitoring probes
7. Rapid cooling until reaching 34℃ (at a maximum speed) and set blanket temperature at least more than 20℃ after the temperature reaches 34℃ to avoid frostbite
8. Mechanical ventilator management: $FiO_2 \leqq 0.6$, $PaO_2 > 100\text{-}150$ torr (mmHg), $PCO_2 = 35\text{-}45$torr (mmHg) (Avoid hyperventilation), respiratory rehabilitation

Supplemental Data: institutional protocol for therapeutic hypothermia –continued 1

9. Sedation

Anaesthesia: i) Midazolam 0.1-0.6 mg/kg/h, ii) Thiamyral/thiopental 1.0-3.0 mg/kg/h

Muscle relaxant for intractable shivering: Vecronium 0.06 mg/kg/h

Analgesia: fentanyl 1-3 μg/kg/h

D. Cooling Phase（Days 2-3）

1. Cardiac function and arrhythmia

Hypothermia prolongs the PR, QRS, QT interval and increases cardiac excitability

arrhythmia other than bradycardia is rare under mild hypothermia

cardiac output decreases with lowering body temperature

2. Blood pressure, heart rate

i) Maintain blood pressure to keep cerebral perfusion pressure (CPP) (= Mean blood pressure [MBP] - ICP) > 50 mmHg

ii) Hypotension: sufficient fluid resuscitation, administration of dopamine or dobutamine

Hypertension: diuretics, Calcium-channel blocker (Nicardipine)

iii) Consider using dobutamine for severe sinus bradycardia

3. Fluid, albumin

i) Fluid resuscitation: 7% acetate ringer solution is recommended.

ii) Avoid hypovolaemia (normal volume replacement): maintain CPP > 50 mmHg

iii) Maintenance fluid should be decreased about 12％ with 1℃ decrease of body temperature and also decreased approximately 15％ with the use of artificial ventilation.

Only 60-70% of the maintenance fluid is normally needed during hypothermia with artificial ventilation.

Supplemental Data: institutional protocol for therapeutic hypothermia –continued 2

4. Electrolyte

i) Serum potassium: control at 3.0-4.5 mEq/L

It decreases in accordance with lower body temperature, and increases in accordance with upper body temperature

ii) Serum sodium: 140-145 mEq/L

iii) Serum glucose: avoid more than 230 mg/dl (may worsen the prognosis)

5. Nutritional Management

Enteral nutrition initiation after day 3 (in cases of severe blood brain barrier damage, early enteral and parenteral nutrition is not recommended).

6. Blood coagulation system

The number of platelet (Plt) decreases with lowering body temperature.

Plt transfusion is not needed when Plt is > 4-5 x 10^4/μL and no bleeding tendency.

The number of Plt increases with body temperature rewarming

PT, APTT may be prolonged with lowering body temperature and recover with rewarming

E. Rewarming Phase（4 days-）

There is no evidence for prolonged hypothermia.

Increase the body temperature at a rate of 0.1℃/h to 36 or 36.5℃

Start weaning sedation gradually when the body temperature reaches 36 or 36.5℃

Extubate when the sedation is fully terminated (Continuous dexmedetomidine infusion may be needed during this period to avoid accidental extubation)

Maintain temperature at 37 ± 0.5℃ for an additional 3 or 4 days

Supplemental Table S1a: Background for each treatment group

	Early-Hypo group (n=8)		Late-Hypo group (n=16)		Non-Hypo group (n=10)	
Age in months, median (IQR)	24.5	(18-35)	17.5	(12.5-23.5)	26.5	(15-48)
Sex, Female, n (%)	5	(62.5)	8	(50)	5	(50)
GCS, median (IQR)	8.5	(7.5*-11.5)	14.5	(13-15)	15	(14-15)
AST, IU/L, median (IQR)	43	(35.5-47)	46.5	(36.5-55.5)	39.5	(37-63)
ALT, IU/L, median (IQR)	15	(12-19)	16.5	(14-27.5)	16.5	(12-24)
LDH, IU/L, median (IQR)	320	(285.5-374)	329.5	(315-387)	334.5	(327-345)
Creatinine, mg/dL, median (IQR)	0.36	(0.27-0.45)	0.29	(0.27-0.32)	0.30	(0.21-0.47)
Blood glucose, mg/dL, median (IQR)	165	(118-237)	228	(146.5-274.5)	165	(118-237)
Duration of seizure in min, median (IQR)	47.5	(30-67.5)	48	(33.5-70)	7.5	(3-59)
Distribution of the lesion Hemisphere, n (%)	2	(25)	7	(43.7)	3	(30)
Bilateral Frontal, n (%)	2	(25)	2	(12.5)	3	(30)
Others, n (%)	4	(50)	7	(43.7)	4	(40)
Diffuse lesions including the perirolandic area, n (%)	1	(12.5)	2	(12.5)	0	(0)
Basal ganglia or thalamus lesion (%)	2	(25)	7	(43.7)	4	(40)
Patients associated with flu infection, n (%)	1	(12.5)	3	(18.8)	4	(40)
Tada score, median (IQR)	5	(3.5-6)	4	(2-5)	2.5	(1-5)
Yokochi score, median (IQR)	3.5	(2.5-5)	3	(0.5-5)	0.5	(0-3)
Patients with biphasic clinical course, n (%)	-	(-)	16	(100)	6	(60)
Duration from 1st seizure to 2nd phase ($Time^{1st\text{-}2nd}$) in hours median (IQR)	-	(-)	88	(71.5-105.4)	114.5	(108-122.5)
Therapeutic options						
Steroid, n (%)	5	(62.5)	6	(37.5)	4	(40)
IVIG , n (%)	2	(25)	4	(25)	2	(20)
Duration, median (IQR)						
1st seizure-cooling initiation ($Time^{1st\text{-}cooling}$)	22.5	(13.2-29.5)	94.3	(89.8-113)	-	(-)
2nd phase-cooling initiation ($Time^{2nd\text{-}cooling}$)	-	(-)	9.5	(5.3-13)	-	(-)
Unfavourable outcome (PCPC > 2), n (%)	5	(62.5)	5	(31.2)	1	(10)

Supplemental Table S1b : Background for each treatment group

	Early-Hypo group (n=8)		Late-Hypo group (n=16)		Non-Hypo group (n=10)	
Worst GCS after 24 hours from 1st seizure	-		10	(7-13.5)	15	(14-15)
Days when reduced subcortical diffusion confirmed on MRI from 1st seizure, median (IQR), n	8	(7-9.5)	4	(4-4.5)	6.5	(5-7)
Days when 1st MRI was taken from 1st seizure median (IQR), n	1	(0.5-1)	3	(1.5-4)	5.5	(2-7)
Reduce subcortical diffusion on first MRI, n (%)	2	(25)	10	(62.5)	6	(60)
Hypotension, n (%)	4	(50)	6	(37.5)	0	(0)
Catecholamine use, n (%)	5	(62.5)	12	(75)	0	(0)
Pneumonia, n (%)	6	(75)	0	(0)	1	(10)
Thrombocytopenia, n (%)	6	(75)	12	(75)	0	(0)
Platelet after 1st seizure	29.2	(18.4-33.6)	27.1	(17.4-33.2)	26.7	(19.5-40.0)
Minimal platelet within 10 days from 1st Seizure	6.0	(4.9-13.4)	12.0	(6.4-15.9)	22.1	(18.2-29.0)
Minimal platelet excluding cooling period	21.5	(17.9-23.3)	16.8	(12.4-21.2)	22.1	(18.2-29.0)
Coagulation disorders, n (%)	8	(100)	14	(87.5)	4	(40)
Arrhythmia, n (%)	0	(0)	0	(0)	0	(0)
Hypokalaemia < 3.5 mEq/L, n (%)	8	(75)	16	(100)	1	(10)
Maximal Creatinine	0.34	(0.28-0.42)	0.32	(0.29-0.36)	0.30	(0.22-0.49)
Dexmedetomidine use during cooling	4	(50)	4	(25)	0	(0)

Supplemental Table S2a: Clinical characteristics of the study population (Patient Number 1-18)

Patient Number	Age (months)	Distribution of the brain lesion on MRI (Hemisphere/ Bilateral frontal lobes/Others)	Diffuse lesions with injury around perirolandic regions (Yes/No)	Basal ganglia /thalamus lesion (Yes/No)	Treatment option (Early-Hypo/Late-Hypo/Non-Hypo group)	Steroid pulse therapy (Yes/No)	IVIG (Yes/ No)	Associated infection	Tada score	BTA timing (days from 1st seizure)	BTA timing: 1, Before 2nd phase; 2, After the initiation of 2nd phase; 3, Absence of 2nd phase	GCS between 12 and 24 h after the first seizure	Worst GCS after 24 h from 1st seizure	Time$_{1st\text{-}cooling}$ (h)
1	31	Others	Yes	Yes	Late-Hypo	Yes	Yes	FluA H3N2	5	4	2	11	3	96.5
2	43	Hemisphere	No	No	Early-Hypo	Yes	No	CoxA9	5	10	3	4		7.5
3	29	Hemisphere	No	No	Late-Hypo	Yes	Yes	FluA H1N1	5	4	2	13	9	90
4	7	Others	No	Yes	Late-Hypo	Yes	No	Unidentified	6	2	2	14	7	64
5	7	Hemisphere	Yes	Yes	Late-Hypo	Yes	Yes	S. pneumoniae meningitis	6	4	2	14	7	91.5
6	12	Hemisphere	No	Yes	Late-Hypo	No	No	Unidentified	3	4	2	15	9	101
7	11	Hemisphere	No	No	Late-Hypo	No	No	Exanthem subitem susp	5	4	2	13	11	92
8	20	Hemisphere	No	No	Late-Hypo	No	No	VZV	3	6	2	15	14	147
9	14	Hemisphere	No	Yes	Late-Hypo	No	No	Unidentified	5	4	2	14	9	110
10	16	Others	No	Yes	Early-Hypo	No	No	Unidentified	5	13	3	8	10	41
11	19	Others	No	No	Late-Hypo	Yes	No	Rota	6	5	2	9	13	116
12	96	Hemisphere	No	No	Non-Hypo	Yes	Yes	FluA	5	5	3	14	14	
13	13	Others	No	No	Late-Hypo	No	No	Exanthem subitem susp	3	4	2	15	6	89.5
14	24	Bilateral frontal lobe	No	No	Early-Hypo	Yes	Yes	Adeno	2	0	3	9		7
15	19	Others	No	No	Late-Hypo	Yes	No	HHV6	0	4	2	15	13	69.5
16	17	Others	No	No	Non-Hypo	Yes	No	VZV	3	7	2	15	15	
17	27	Others	No	No	Early-Hypo	Yes	No	Unidentified	7	8	3	8		19
18	9	Others	No	No	Early-Hypo	Yes	Yes	Unidentified	3	8	3	15	13	25

IVIG, intravenous immunoglobulin; BTA, bright tree appearance on diffusion weighted image; GCS, Glasgow Coma Scale; Flu, Influenza virus; Cox, Coxsackievirus; VZV, varicella-zoster virus; HHV6, Human herpes virus 6; Time$_{1st\text{-}cooling}$; duration between the first seizure and the initiation of therapeutic hypothermia

Supplemental Table S2b: Clinical characteristics of the study population (Patient Number 1-18)

Patient Number	Hypotension at admission (Yes/No)	Hypotension within 10 days (Yes/No)	CA at admission (Yes/No)	CA within 10 days (Yes/No)	Pneumonia (Yes/No)	Plt count (x10^3/μL) at 1st seizure	Min Plt (x10^3/μL) within 10 days	Min Plt within 10 days excluding cooling period	PT before cooing (s)	PT INR before cooling	APTT before cooling (s)	Max PT (s)	Max PT INR	Max APTT (s)	K< 3.5 (mEq/L) at admission (Yes/No)	K<3.5 (mEq/L) within 10 days (Yes/No)	Max creatinine (mg/dL)	DEX use during cooling (μg/kg/h)
1	No	Yes	No	No	No	26	17	21.0	15.0	1.23	42.4	15	1.23	42.4	No	Yes	0.37	No
2	No	No	No	Yes	Yes	33.5	24.1	32.4	14.4	1.20	34.9	14.6	1.22	40.5	No	Yes	0.43	No
3	No	No	No	Yes	No	32.3	10.5	16.3				11.1	0.91	31.9	No	Yes	0.35	No
4	No	No	No	Yes	No	44.0	7.1	18.2				11.8	1.03	51	No	Yes	0.32	No
5	No	No	No	Yes	No	38.7	26.5	27.7	17.6	1.51	39.7	20.1	1.73	79.7	No	Yes	0.38	No
6	No	No	No	Yes	No	30.7	11.9	19.7	9.6	0.90	36	13	1.22	47.9	No	Yes	0.40	No
7	No	No	No	Yes	No	15.6	14.8	15.6				14.1	1.19	47.2	No	Yes	0.32	No
8	No	Yes	No	Yes	No	39.4	26.4	26.4				13.3	1.13	46.5	No	Yes	0.28	No
9	No	No	No	Yes	No	28.6	19.7	21.4	12.9	1.09	30.1	17.6	1.49	55.3	No	Yes	0.32	0.5
10	No	No	No	Yes	Yes	28.1	5.8	22.1				15.6	1.32	153.1	Yes	Yes	0.29	0.3-0.7
11	No	No	No	Yes	No	34.1	4.5	4.5	17.4	1.46	46.4	17.4	1.46	57.8	No	Yes	0.37	0.5-0.6
12	No	No	No	No	Yes	23.6	16.7	16.7				16.3	1.34	36.5	No	Yes	0.68	
13	No	No	No	Yes	No	27.4	6.5	9.6	12.9	1.09	26.8	13.5	1.16	61	No	Yes	0.29	0.7
14	No	Yes	No	No	No	30.3	6.1	20.9	14.2	1.17	39.2	17.9	1.44	162.7	No	Yes	0.18	0.7
15	No	No	No	No	No	16.4	6.3	12.6	12.8	1.07	58.9	14.1	1.17	61.9	Yes	Yes	0.26	0.2-0.5
16	No	No	No	No	No	14.2	14.2	14.2				12.5	1.05	38.4	No	No	0.26	
17	No	Yes	No	Yes	Yes	36.0	4.2	23.1				17.4	1.24	93.6	No	Yes	0.46	0.5-0.9
18	No	No	No	No	No	19.1	3.5	18.2	15.5	1.30	49.1	17.4	1.45	100	No	Yes	0.27	0.5-0.7

CA, catecholamine use ; Plt, platelet; PT, prothrombin; PT INR, prothrombin international normalised ratio; APTT, activated partial thromboplastin time; K, potassium; DEX, dexmedetomidine

Supplemental Table S2c: Clinical characteristics of the study population (Patient Number 1-18)

Patient Number	1st CSF Cell (/µL) (lymphocytes, %)	Protein (mg/dL)	Timing of 1st CSF from 1st seizure (day)	2nd CSF Cell (/µL) (lymphocytes, %)	Protein (mg/dL)	Timing of 2nd CSF from 1st seizure (day)
1	1 (100)	9	0			
2	1 (67)	26	0			
3	1 (0)	18	0	15 (73)	58	4
4	1 (100)	30	0	12 (100)	17	2
5	2704 (4)	173.2	0	1365 (12)	84.8	2
6	2 (100)	17.7	1			
7						
8	4 (82)	12.3	6			
9	1 (100)	31.9	0			
10	1 (100)	15.2	2			
11	<1 (100)	14	0			
12						
13	<1 (100)	20	1			
14	4 (100)	14	0			
15	1 (100)	24	4			
16	3 (100)	19	6			
17	1 (100)	16	1			
18	1 (100)	51	0			

CA, catecholamine use ; Plt, platelet; PT, prothrombin; PT INR, prothrombin international normalised ratio; APTT, activated partial thromboplastin time; K, potassium; DEX, dexmedetomidine

Supplemental Table S2d: Clinical characteristics of the study population (Patient Number 1-18) –continued

Patient Number	Age (months)	Distribution of the brain lesion on MRI (Hemisphere/ Bilateral frontal lobes/Others)	Diffuse lesions with injury around perirolandic regions (Yes/No)	Basal ganglia /thalamus lesion (Yes/No)	Treatment option (Early-Hypo/Late-Hypo/Non-Hypo group)	Steroid pulse therapy (Yes/No)	IVIG (Yes/ No)	Associated infection	Tada score	BTA timing (days from 1st seizure)	BTA timing: 1, Before 2nd phase; 2, After the initiation of 2nd phase; 3, Absence of 2nd phase	GCS between 12 and 24 h after the first seizure	Worst GCS after 24 h from 1st seizure	$Time_{1st\text{-}cooling}$ (h)
19	32	Others	No	Yes	Non-Hypo	Yes	No	Unidentified	1	10	2	15	15	
20	13	Hemisphere	No	Yes	Non-Hypo	No	No	Exanthem subitem susp	1	7	2	15	15	
21	21	Bilateral frontal lobe	No	No	Non-Hypo	Yes	Yes	Unidentified	3	5	2	15	15	
22	25	Others	No	No	Early-Hypo	Yes	No	Unidentified	6	6	3	11		20
23	39	Others	No	Yes	Non-Hypo	No	No	Unidentified	2	6	3	13	14	
24	21	Bilateral frontal lobe	No	No	Late-Hypo	No	No	Unidentified	0	5	2	15	14	124
25	15	Others	No	Yes	Non-Hypo	No	No	Unidentified	1	7	2	15	15	
26	15	Others	No	Yes	Late-Hypo	No	Yes	Kawasaki disease	5	3	2	11	4	74
27	11	Bilateral frontal lobe	No	No	Non-Hypo	No	No	FluA	2	6	2	15	15	
28	71	Hemisphere	No	No	Non-Hypo	No	No	FluB	6	8	3	14	14	
29	48	Bilateral frontal lobe	No	No	Non-Hypo	No	No	FluA	5	5	3	11	13	
30	117	Hemisphere	Yes	Yes	Early-Hypo	No	No	Flu B	4	9	3	12	9	33
31	39	Hemisphere	No	No	Late-Hypo	No	No	FluA H1N1	1	5	2	15	15	127
32	20	Bilateral frontal lobe	No	No	Early-Hypo	No	No	Unidentified	6	8	3	7	7	26
33	26	Bilateral frontal lobe	No	Yes	Late-Hypo	No	No	Unidentified	2	4	2	15	13	104
34	16	Others	No	No	Late-Hypo	No	No	HHV6	2	4	2	15	14	91.5

IVIG, intravenous immunoglobulin; BTA, bright tree appearance on diffusion weighted image; GCS, Glasgow Coma Scale; Flu, Influenza virus; HHV6, Human herpes virus 6; $Time_{1st\text{-}cooling}$; duration between the first seizure and the initiation of therapeutic hypothermia

Supplemental Table S2e: Clinical characteristics of the study population (Patient Number 1-18)

Patient Number	Hypotension at admission (Yes/No)	Hypotension within 10 days (Yes/No)	CA at admission (Yes/No)	CA within 10 days (Yes/No)	Pneumonia (Yes/No)	Plt count ($x10^3/μL$) at 1st seizure	Min Plt ($x10^3/μL$) within 10 days	Min Plt within 10 days excluding cooling period	PT before cooing (s)	PT INR before cooling	APTT before cooling (s)	Max PT (s)	Max PT INR	Max APTT (s)	K< 3.5 (mEq/L) at admission (Yes/No)	K<3.5 (mEq/L) within 10 days (Yes/No)	Max creatinine (mg/dL)	DEX use during cooling (μg/kg/h)
19	No	No	No	No	No	28.7	24.9	24.9							No	No	0.22	
20	No	No	No	No	No	19.5	19.5	19.5							No	No	0.28	
21	No	No	No	No	No	18.2	18.2	18.2				11.5	0.98	30.2	No	No	0.36	
22	No	No	No	No	Yes	33.7	19.4	23.5				16.3	1.28	37.2	No	Yes	0.4	No
23	No	No	No	No	No	40	29	29				12.8	1.02	23.2	No	No	0.32	
24	No	Yes	No	No	No	26.7	14.8	17.2	12.3	0.98	32.5	12.3	0.98	32.5	No	Yes	0.27	No
25	No	No	No	No	No	40.8	30	30				12.6	1.07	34.5	No	No	0.22	
26	No	No	No	Yes	No	16.7	2.4	5.4	12.9	1.1	35	14.2	1.2	58.9	No	Yes	0.31	No
27	No	No	No	No	No	24.7	24.7	24.7							No	No	0.21	
28	No	No	No	No	No	37.3	19.4	19.4				13.8	1.17	28	No	No	0.61	
29	No	No	No	No	No	43.2	30.8	30.8				16.5	1.39	37.1	No	No	0.49	
30	No	Yes	No	Yes	Yes	22.5	5.5	17.6	13.7	1.16	38.5	15.3	1.29	52.3	No	Yes	0.36	No
31	No	Yes	No	No	No	26.5	24.1	24.1	11.5	0.98	33	13.3	1.13	42.1	No	Yes	0.32	No
32	No	Yes	No	Yes	Yes	16.8	7.4	16.8	15	1.31	38.4	17.1	1.5	68.3	No	Yes	0.32	No
33	No	Yes	No	Yes	No	12.6	14	12.6	12.1	1.05	34.9	14.2	1.24	37.8	No	Yes	0.29	No
34	No	Yes	No	Yes	No	18	6.2	12.1	10.8	0.94	32.4	14.4	1.26	41.3	Yes	Yes	0.25	No

CA, catecholamine use ; Plt, platelet; PT, prothrombin; PT INR, prothrombin international normalised ratio; APTT, activated partial thromboplastin time; K, potassium; DEX, dexmedetomidine

Supplemental Table S2f: Clinical characteristics of the study population (Patient Number 1-18)

Patient Number	1st CSF Cell (/µL) (lymphocytes, %)	Protein (mg/dL)	Timing of 1st CSF from 1st seizure (day)	2nd CSF Cell (/µL) (lymphocytes, %)	Protein (mg/dL)	Timing of 2nd CSF from 1st seizure (day)
19	<1 (100)	16	10			
20						
21	1 (100)	14	0			
22	3 (100)	25.4	0			
23	<1 (100)	15	6			
24						
25	9 (100)	20	7			
26						
27	1 (100)	29	5			
28	<1 (100)	19	1			
29	1 (100)	19	9			
30	2 (100)	23	1			
31						
32	4 (100)	21	0			
33	2 (100)	24.6	4			
34	8 (96)	38	4			

CA, catecholamine use ; Plt, platelet; PT, prothrombin; PT INR, prothrombin international normalised ratio; APTT, activated partial thromboplastin time; K, potassium; DEX, dexmedetomidine

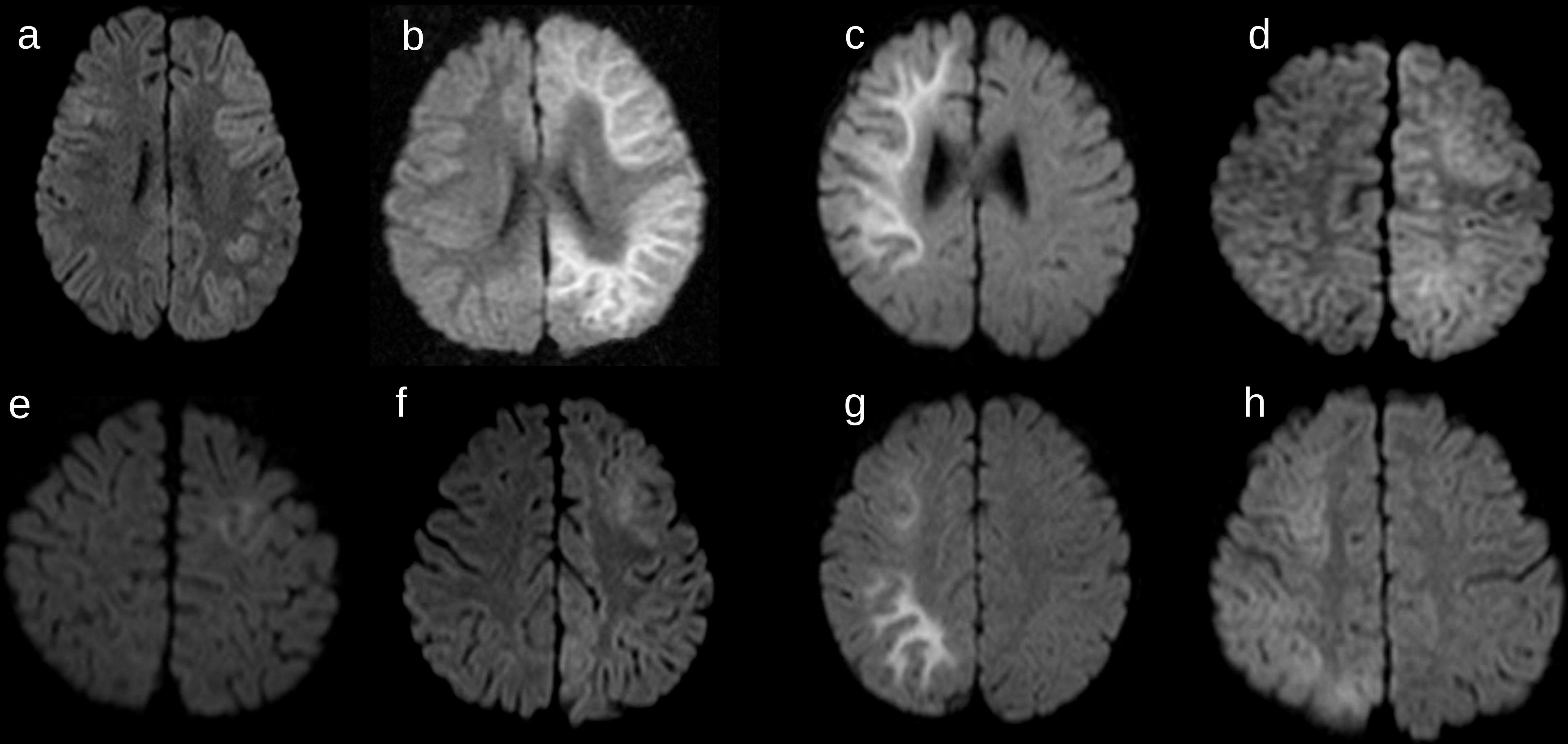
a
b
c
d
e
f
g
h

Supplemental Figure S1 -continued

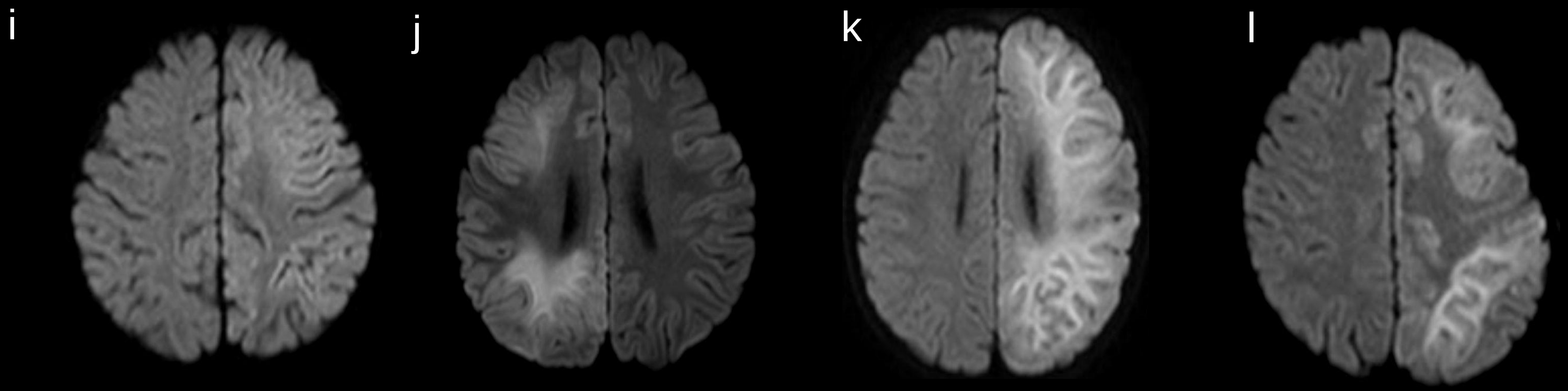

Fig. S1. Magnetic resonance imaging with hemisphere lesions (DWI). Twelve patients showed hemisphere lesions: a, Patient No. 2. b, Patient No. 3. c, Patient No. 5. d, Patient No. 6. e, Patient No. 7. f, Patient No. 8 g, Patient No. 9. h, Patient No. 12. i, Patient No. 20. j, Patient No. 28. k, Patient No. 30. l, Patient No. 31.
DWI, Diffusion-weighted imaging

Supplemental Figure S2

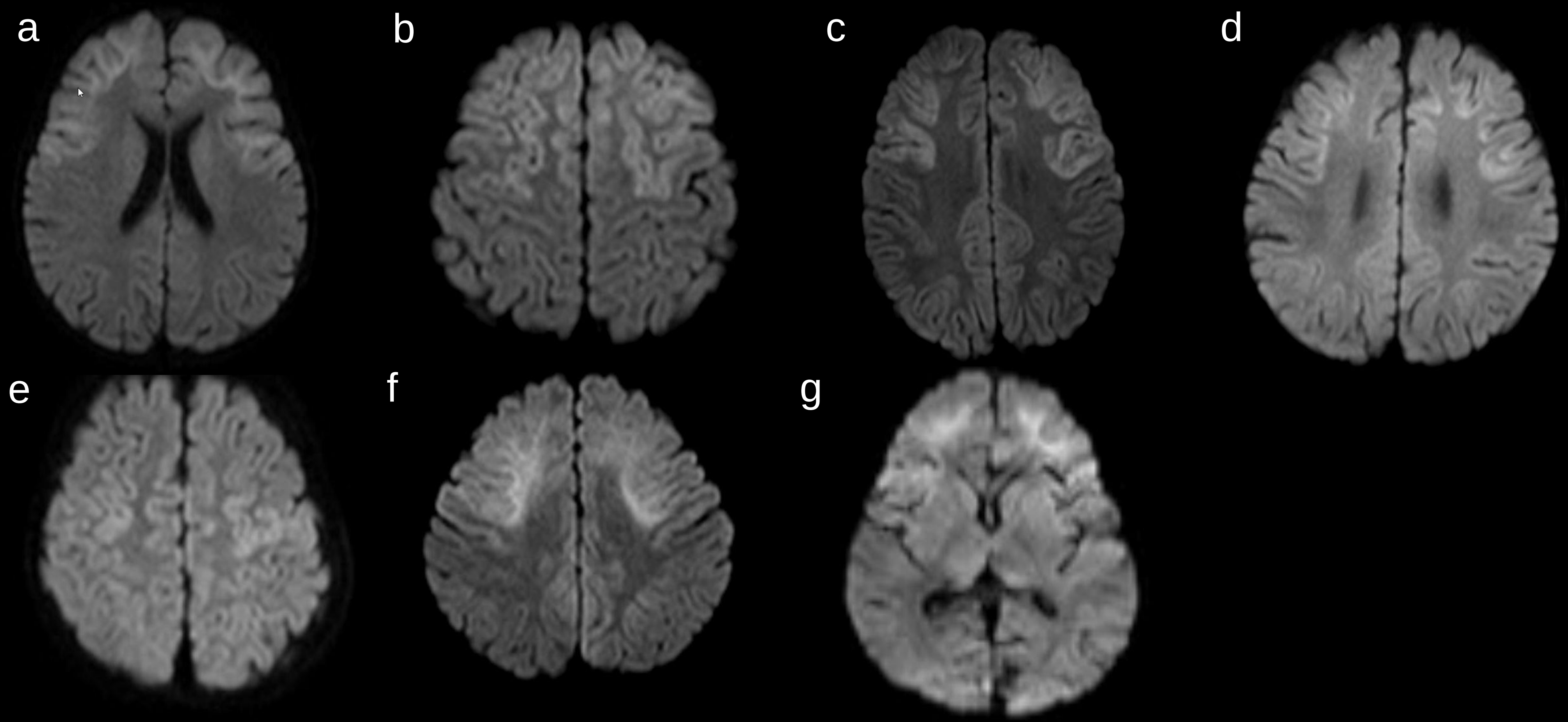

Fig. S2. Magnetic resonance imaging with bilateral frontal lobe lesions (DWI). Seven patients showed bilateral frontal lobe lesions: a, Patient No. 14. b, Patient No. 21. c, Patient No. 24. d, Patient No. 27. e, Patient No. 29. f, Patient No. 32. g, Patient No. 33.

DWI, Diffusion-weighted imaging.

Supplemental Figure S3

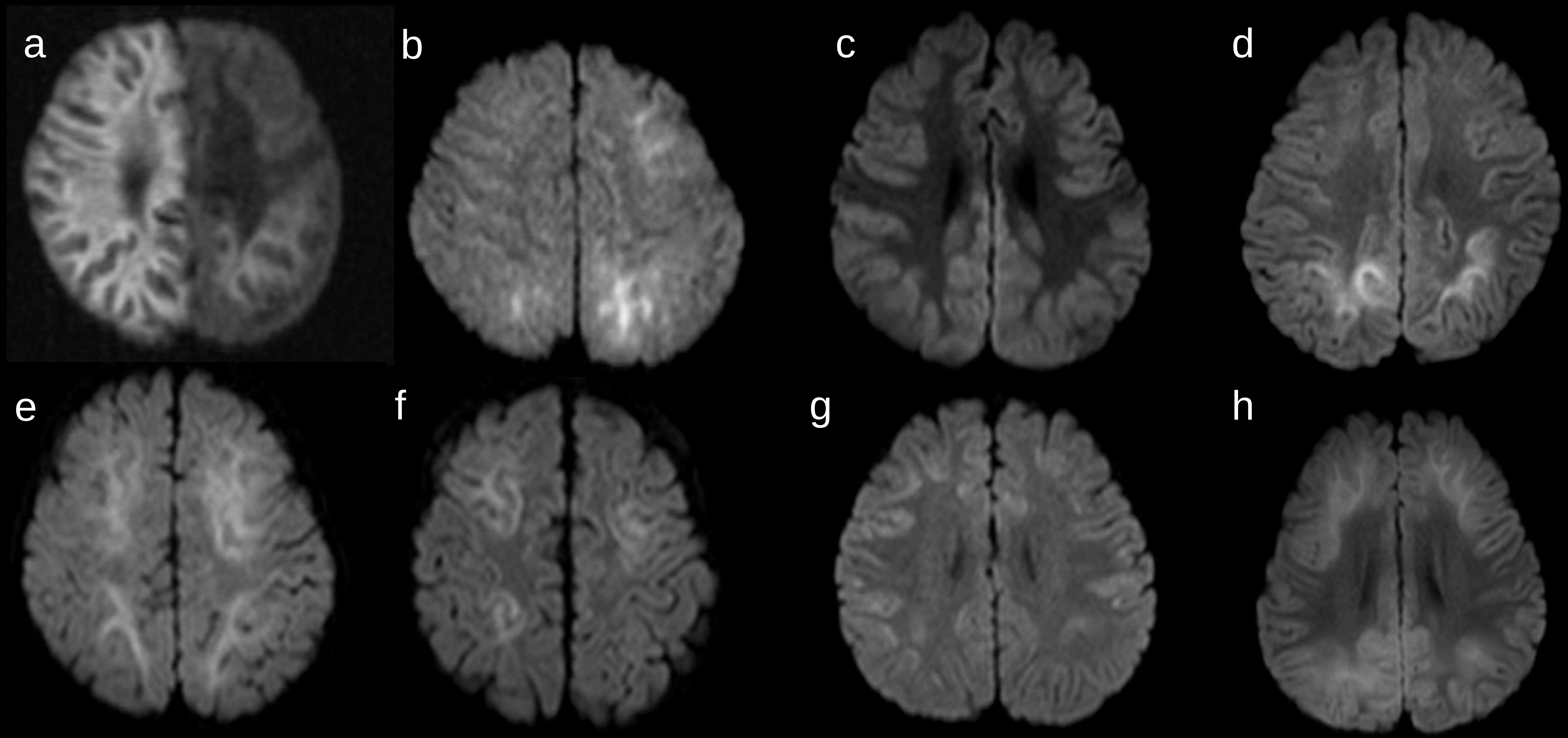

Supplemental Figure S3 -continued

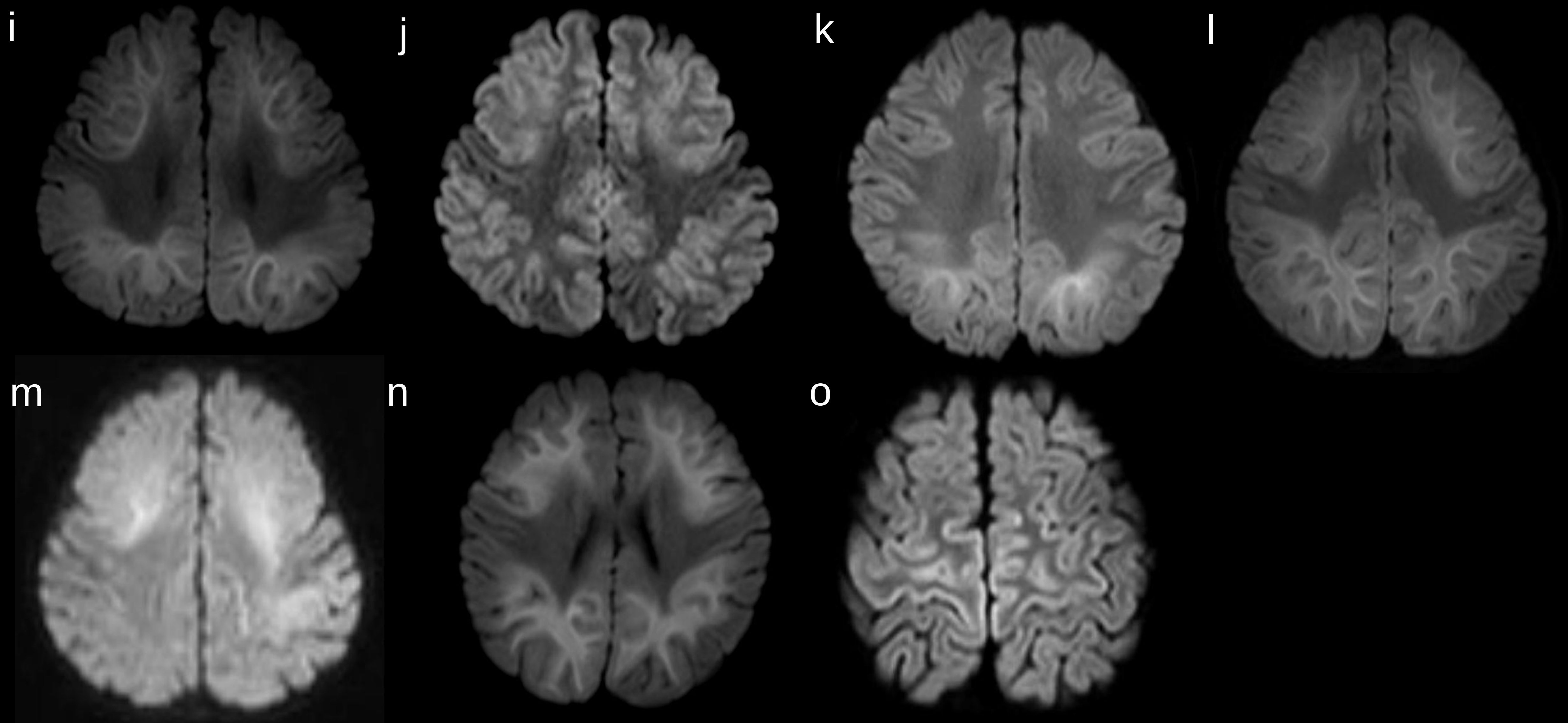

Fig. S3. Magnetic resonance imaging with other distribution types of injuries (DWI): a, Patient No. 1. b, Patient No. 4. c, Patient No. 10. d, Patient No. 11. e, Patient No. 13. f, Patient No. 15. g, Patient No. 16. h, Patient No. 17. i, Patient No. 18. j, Patient No. 19. k, Patient No. 22. l Patient No. 23. m, Patient No. 25. n, Patient No. 26. o, Patient No. 34.
DWI, Diffusion-weighted imaging

Supplemental Figure S4

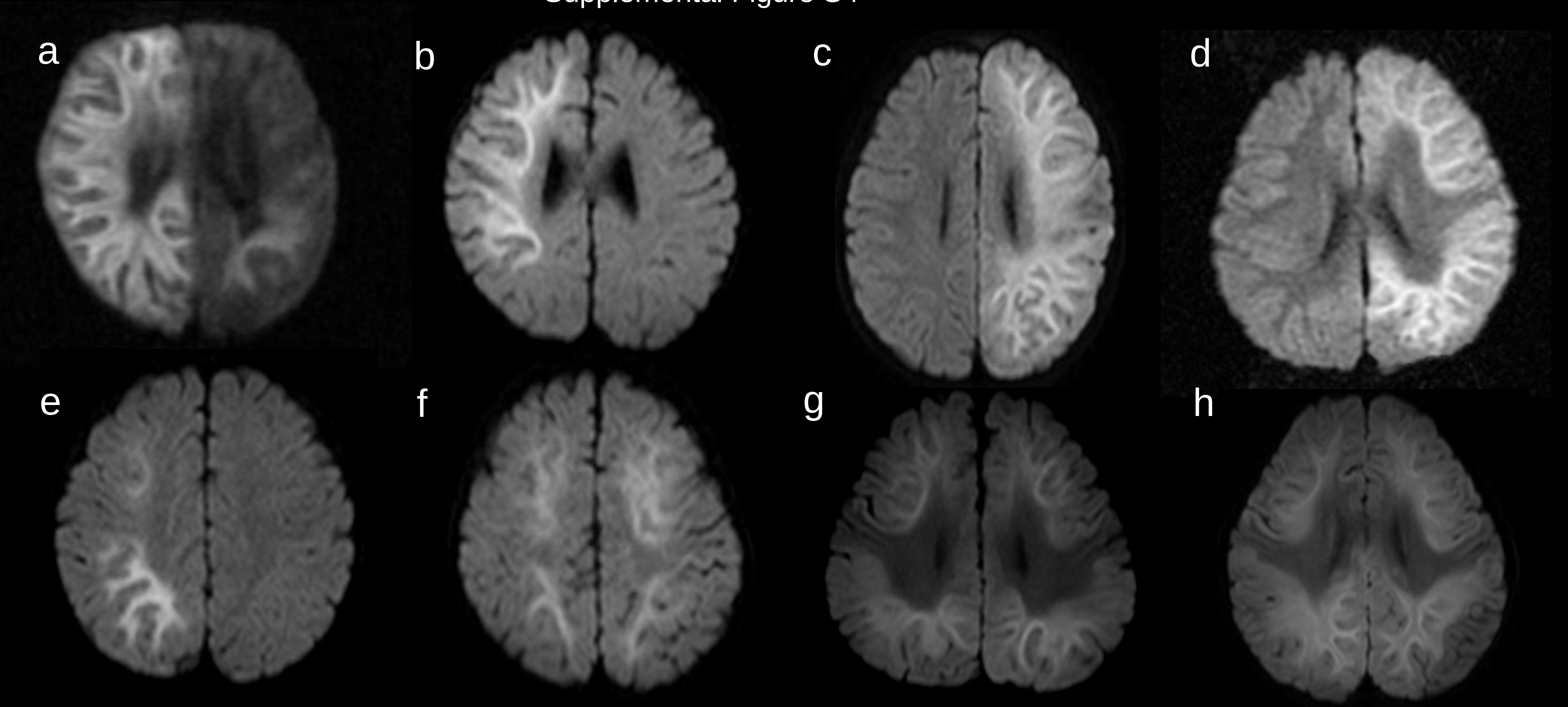

Fig. S4. Diffuse lesions with injury around perirolandic regions (a-c) and magnetic resonance imaging without injury around perirolandic regions (d-h) on DWI. a, Patient No. 1. b, Patient No. 5. c, Patient No. 30. Example of magnetic resonance imaging with central-sparing in five patients: d, Patient No. 3. e, Patient No. 9. f, Patient No. 13. g, Patient No. 18. h, Patient No. 23. DWI, Diffusion-weighted imaging

Supplemental Figure S5

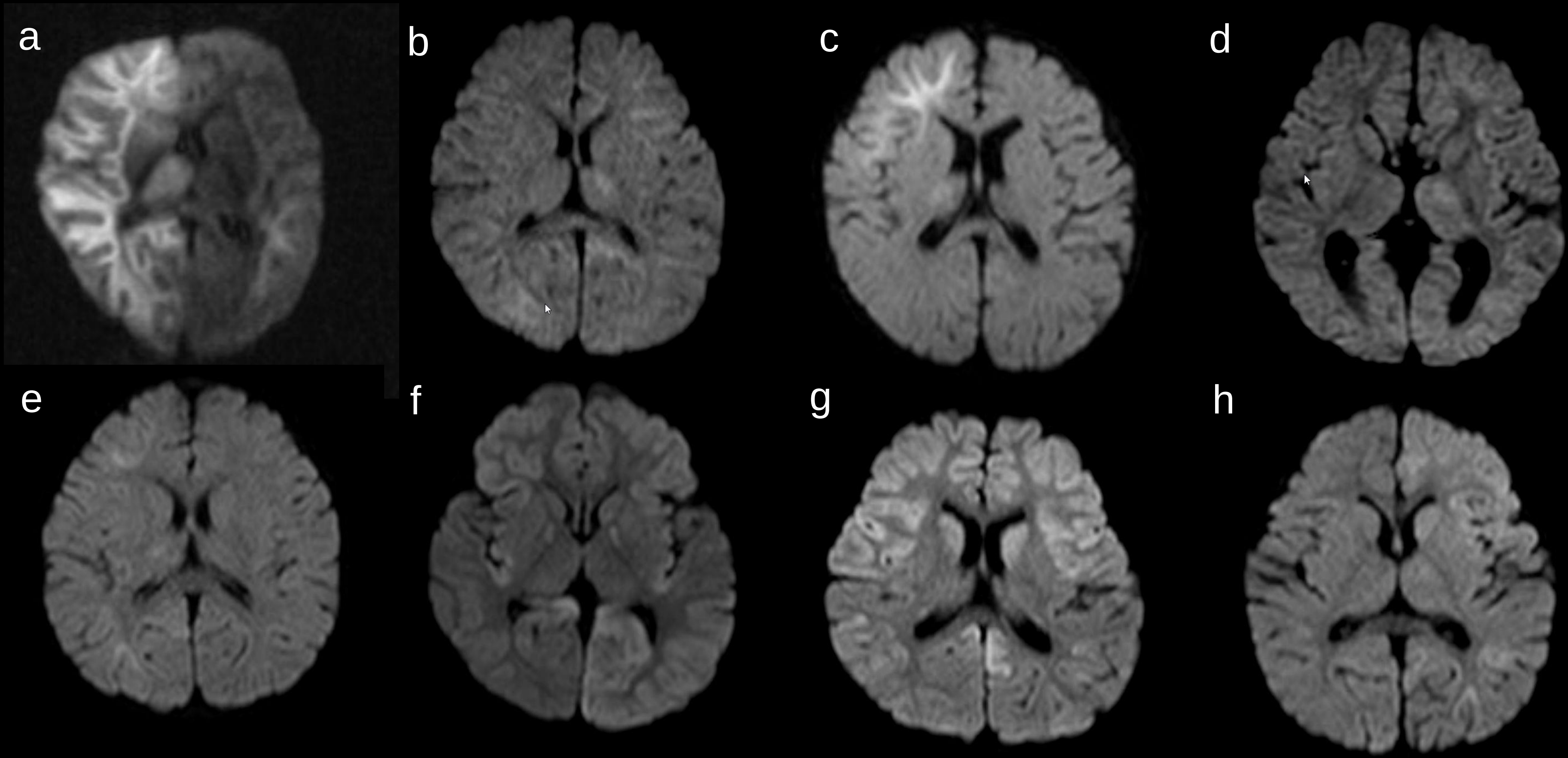

Supplemental Figure S5 -continued

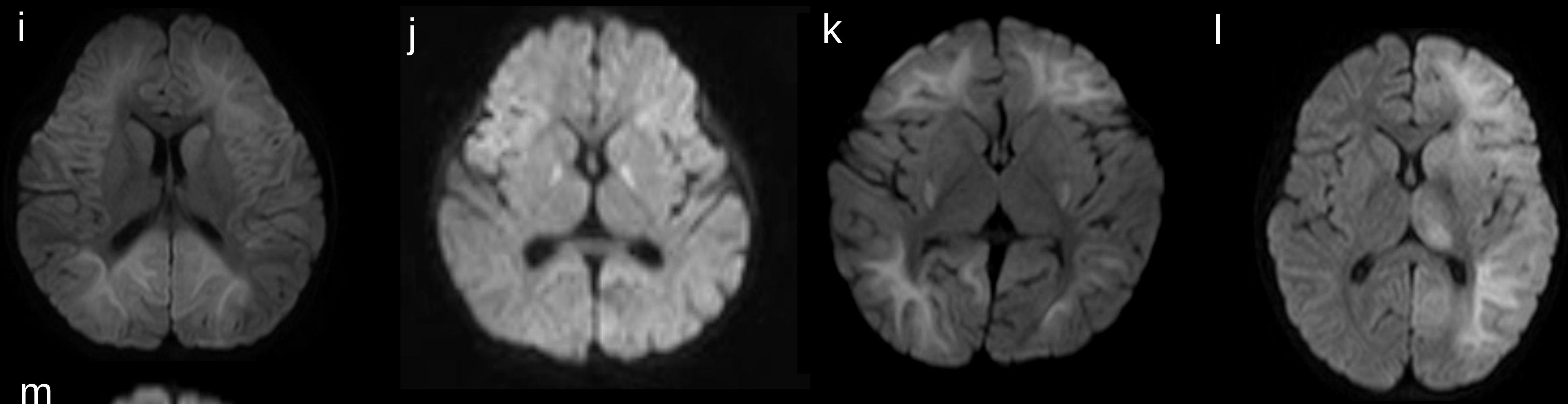

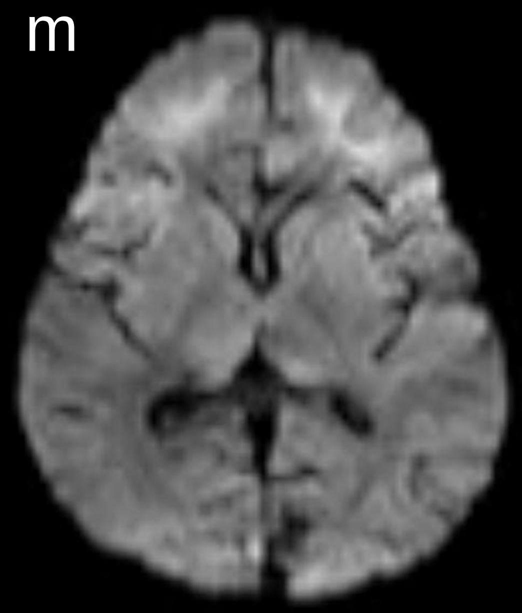

Fig. S5. Magnetic resonance imaging with basal ganglia or thalamus lesion (DWI).
Thirteen patients showed basal ganglia or thalamus lesions: a, Patient No. 1. b, Patient No. 4. c, Patient No. 5. d, Patient No. 6. e, Patient No. 9. f, Patient No. 10. g, Patient No. 19. h, Patient No. 20. i, Patient No. 23. j, Patient No. 25. k, Patient No. 26. l, Patient No. 30. m. Patient No. 33.
DWI, Diffusion-weighted imaging

Supplemental Figure S6

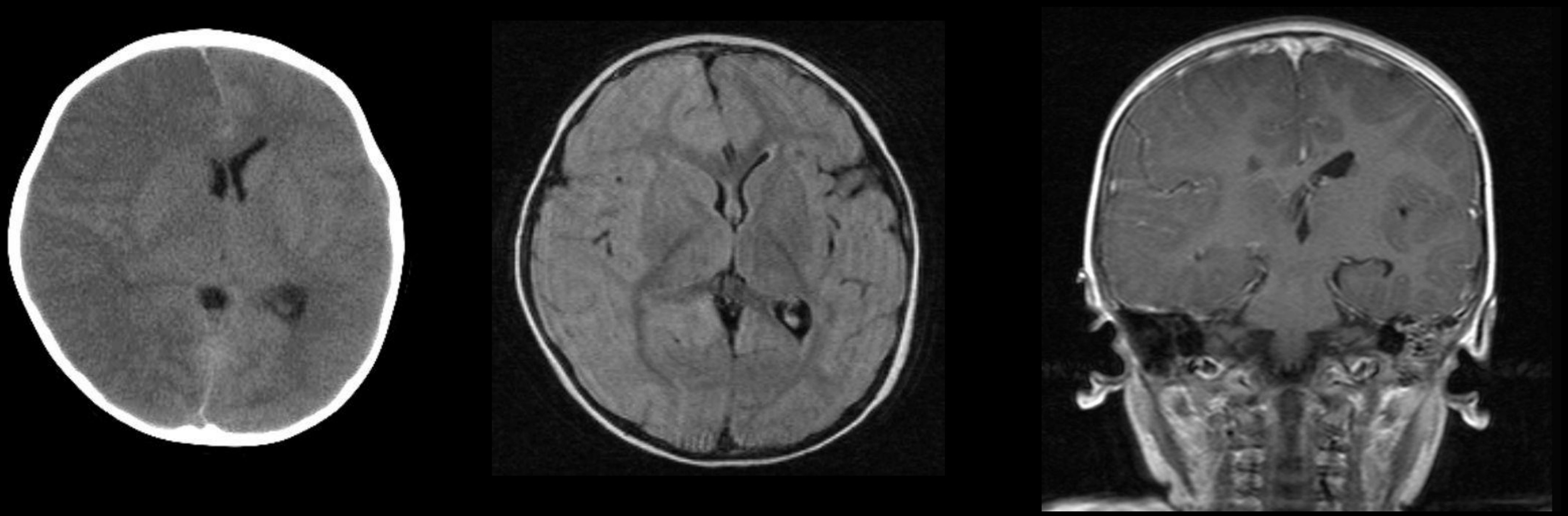

Fig. S6. Neuroimaging (a, Axial head CT scan. b, Axial FLAIR image. c, Coronal T1-weighted image) showed prominent midsagittal line shift with symptoms, such as anisocoria, coma, at the beginning of the second phase (Patient No. 1).
CT, computed tomography; FLAIR, fluid-attenuated inversion-recovery

Supplemental Figure S7

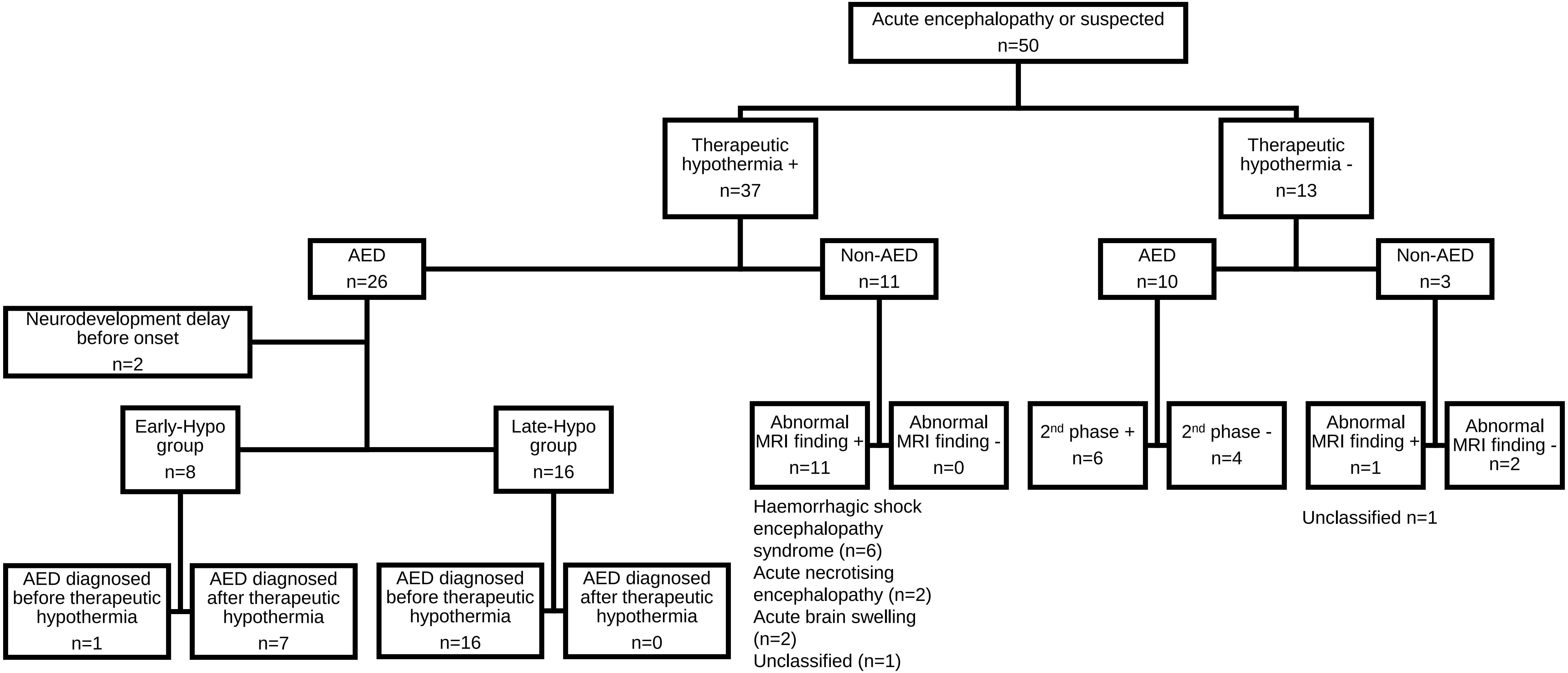

Fig. S7. Among 50 children with or suspected acute encephalopathy, 37 patients were provided therapeutic hypothermia, among whom 26 patients were diagnosed as having AED. Thirteen patients were not provided therapeutic hypothermia, among whom 10 patients were diagnosed as ASD. After excluding two patients with AED with neurodevelopment delay before onset, 34 patients were retrospectively enrolled in this study.

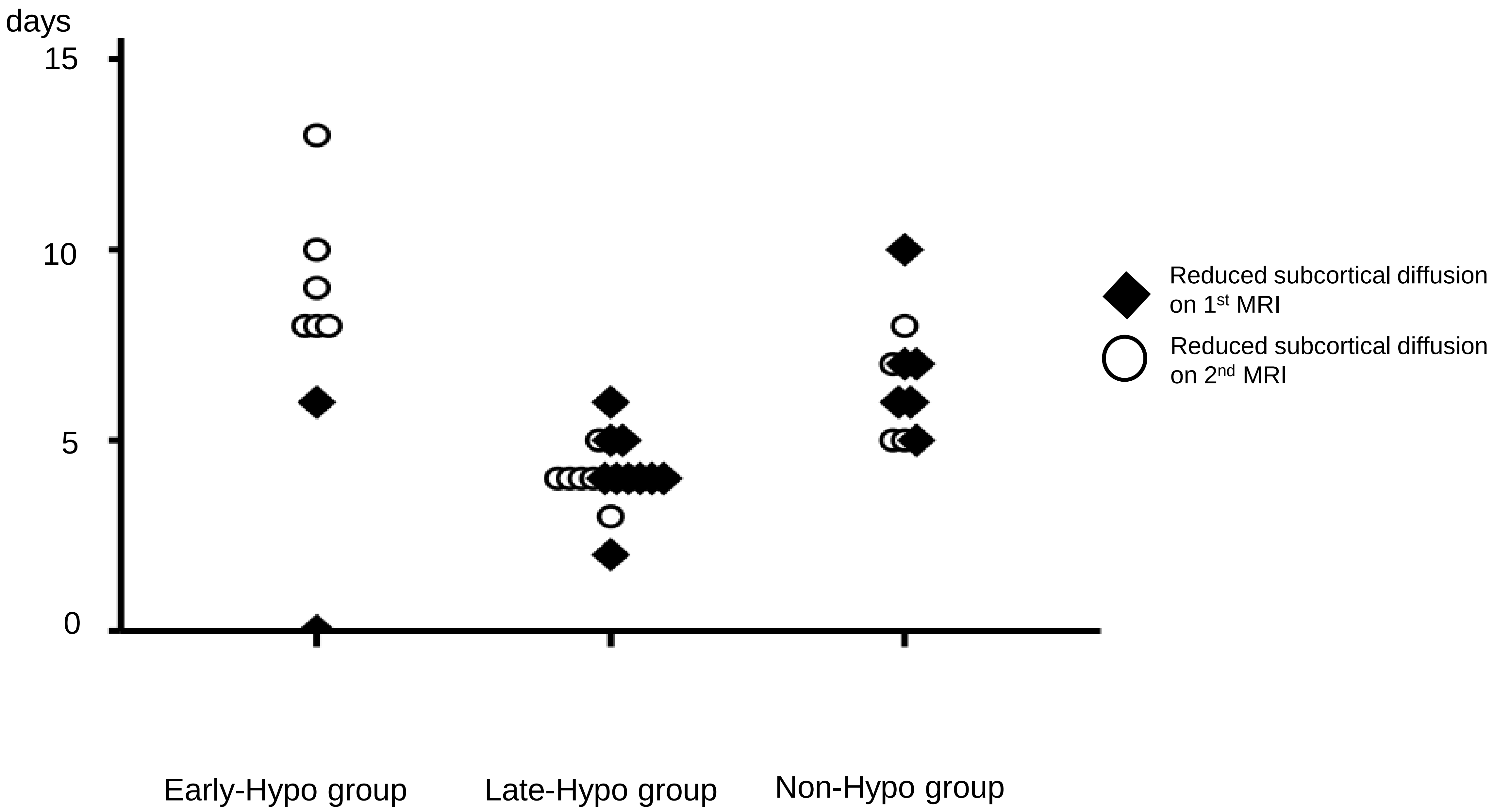

Fig. S8. Days when reduced subcortical diffusion confirmed on MRI from 1st seizure

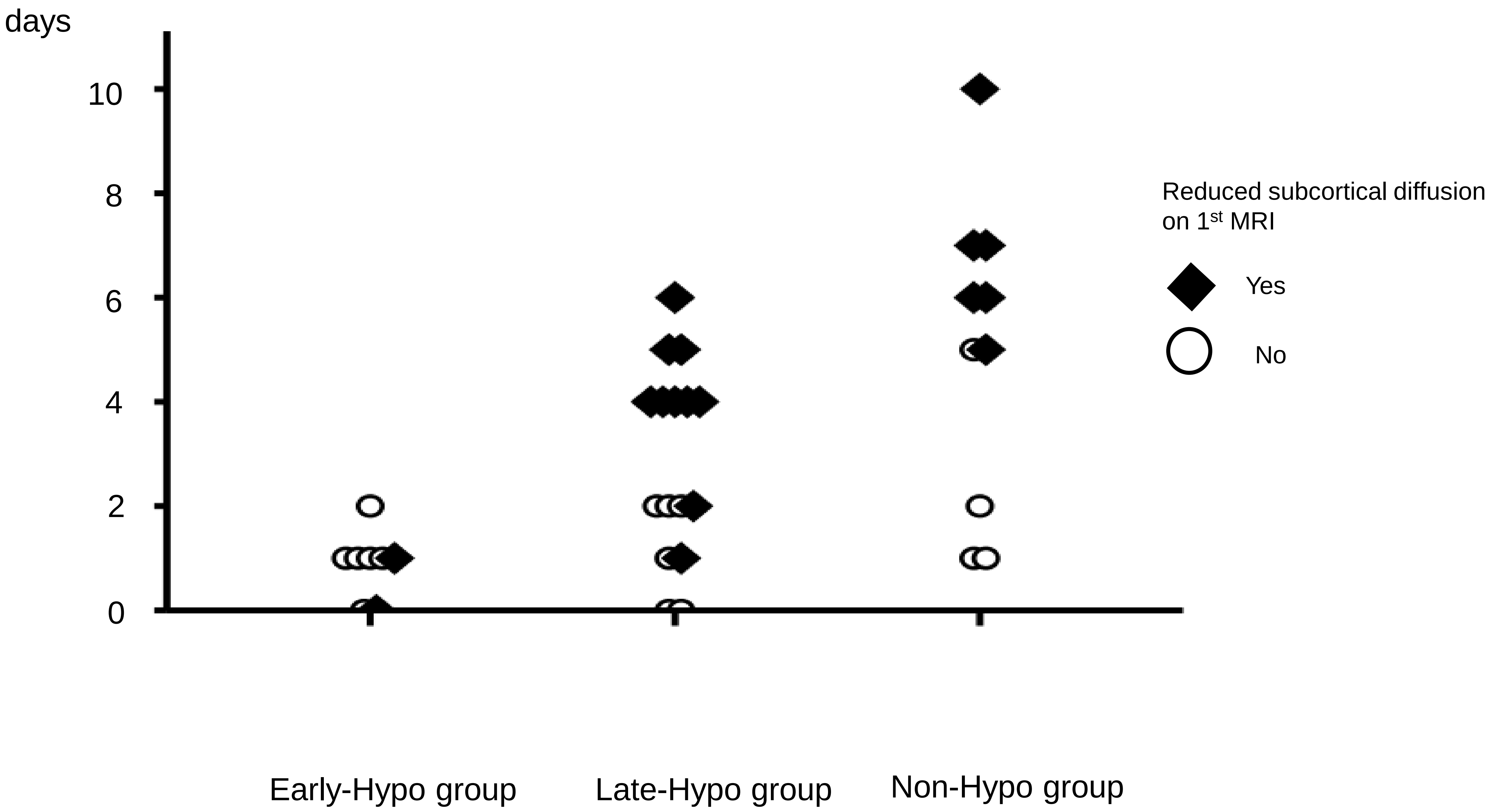

Fig. S9. Days when 1st MRI was taken from 1st seizure

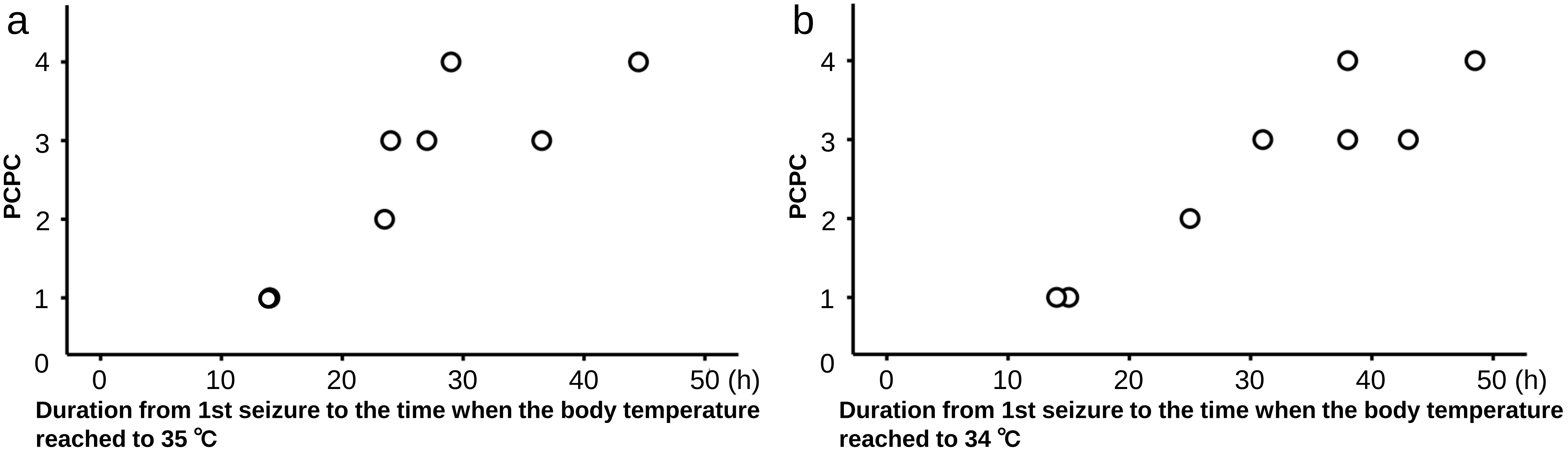

Fig. S10. The relationship between the outcome represented by the Paediatric Performance Category Scale (PCPC) after 1 year and the duration from the first seizure to the time when the body temperature reached 35℃ (a) or 34℃ (b) in the Early-Hypo group.

The outcome is dependent on the timing of the duration from the first seizure to the time when the body temperature reached at 35℃ (A, $r = 0.907$, $p = 0.002$, Spearman's rank correlation coefficient) or 34℃ (B, $r = 0.876$, $p = 0.004$, Spearman's rank correlation coefficient) These were statistically significant after adjusted by the Benjamini–Hochberg procedure to correct multiple comparisons, using a false discovery rate of 0.05.

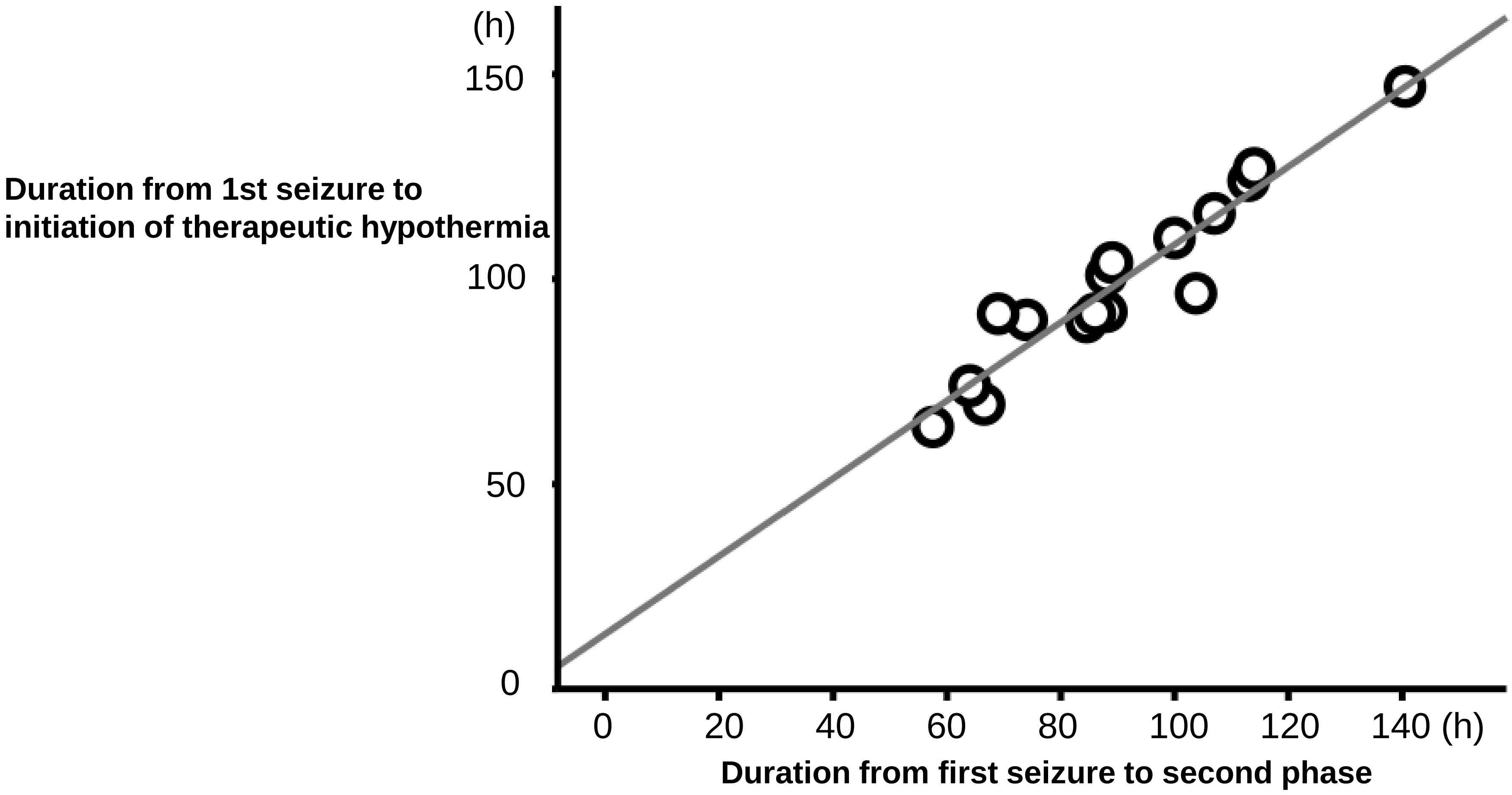

Fig. S11. The relationship between the duration from the 1st seizure to the initiation of the 2nd phase and the duration from the 1st seizure to the initiation of therapeutic hypothermia in the Late-Hypo group.
There was a significant association observed between those two (n = 16, r = 0.954, r < 0.001, Pearson's correlation coefficient). These were statistically significant after adjusted by the Benjamini–Hochberg procedure to correct multiple comparisons, using a false discovery rate of 0.05.

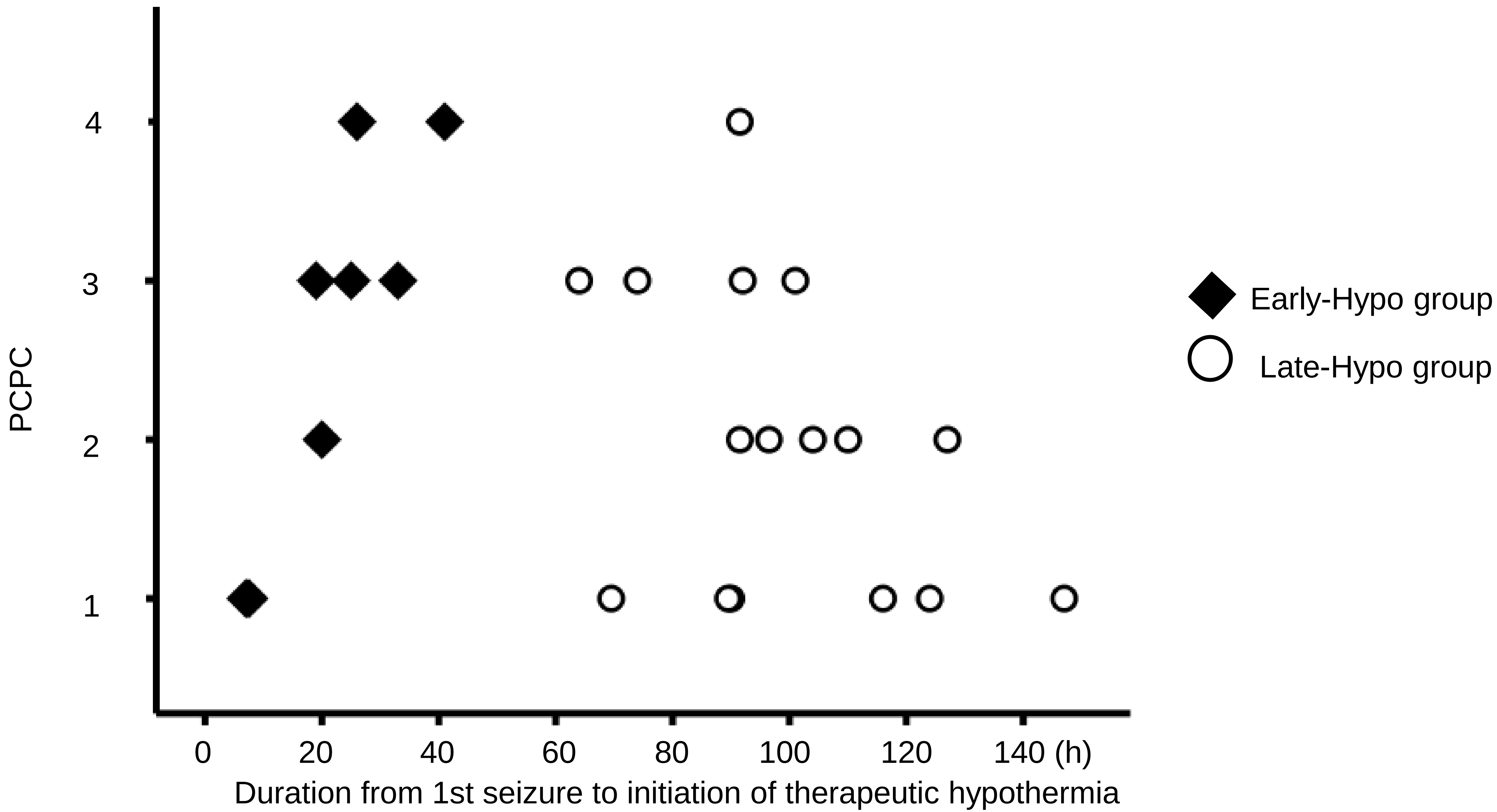

Fig. S12. The relationship between outcomes and the duration from the 1st seizure to the initiation of therapeutic hypothermia (Time$_{1st\text{-}cooling}$) in whole patients with AED. All patients in the Early-Hypo group had therapeutic hypothermia initiated at 60 h prior to the first seizure.

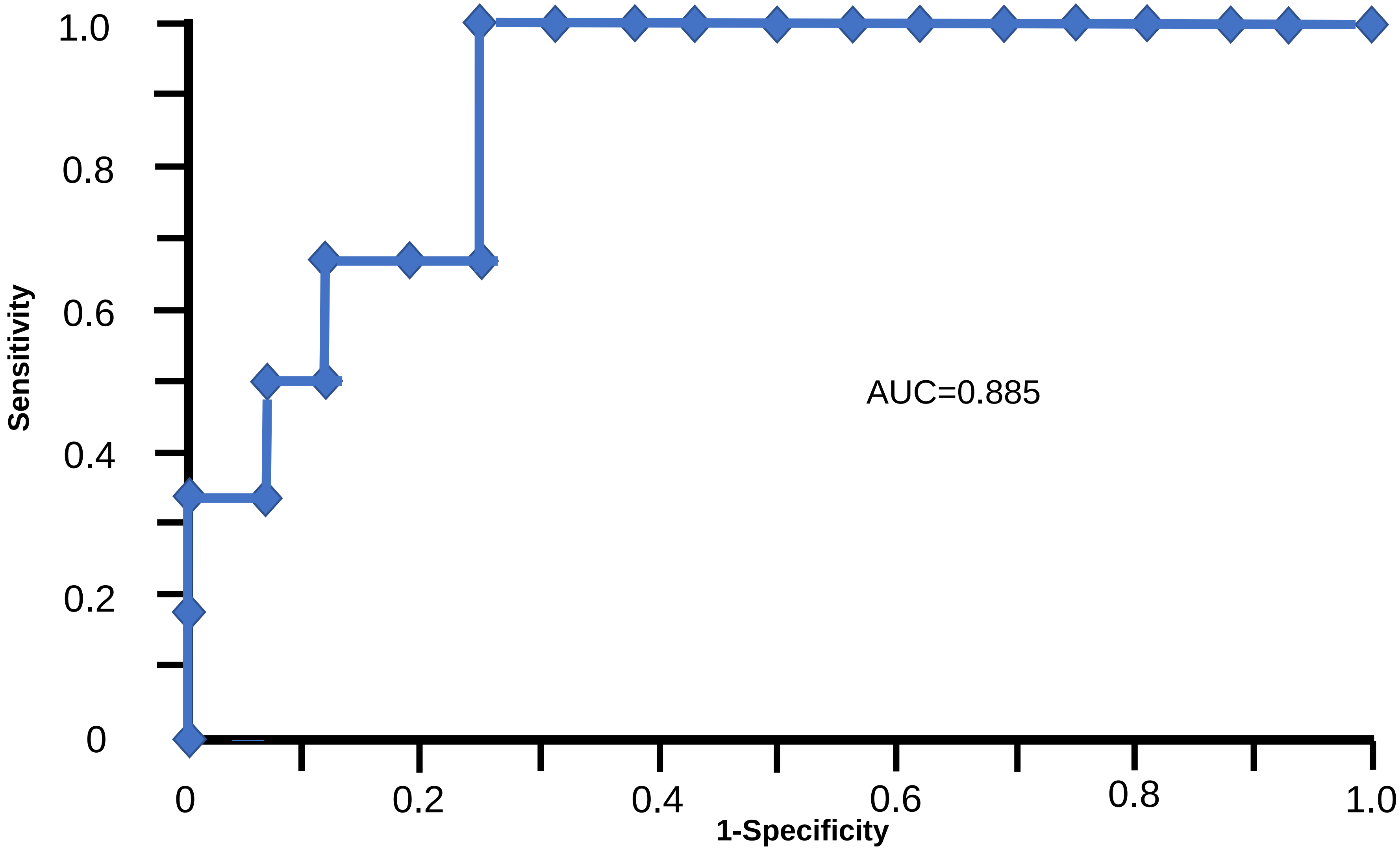

Fig. S13. Receiver operating characteristic (ROC) curve of the duration from the 1st seizure to the initiation of the 2nd phase in patients with AED with biphasic clinical course for prediction of unfavourable outcome.
The cut-off value determined from the ROC curve was 83.75 h. The sensitivity was 66.7% and the specificity was 87.5%.
AUC, area under the curve
